# Highly efficient and stable perovskite solar cells enabled by low-dimensional perovskitoids

**DOI:** 10.1126/sciadv.abk2722

**Published:** 2022-01-26

**Authors:** Jinbo Chen, Yingguo Yang, Hua Dong, Jingrui Li, Xinyi Zhu, Jie Xu, Fang Pan, Fang Yuan, Jinfei Dai, Bo Jiao, Xun Hou, Alex K.-Y. Jen, Zhaoxin Wu

**Affiliations:** 1 Key Laboratory for Physical Electronics and Devices of the Ministry of Education & Shaanxi Key Lab of Information Photonic Technique, School of Electronic Science and Engineering, Xi’an Jiaotong University, Xi’an, Shaanxi 710049, China.; 2 Shanghai Synchrotron Radiation Facility (SSRF), Zhangjiang Lab, Shanghai Advanced Research Institute, Shanghai Institute of Applied Physics, Chinese Academy of Science, Shanghai 201204, China.; 3 Collaborative Innovation Center of Extreme Optics, Shanxi University, Taiyuan, Shanxi 030006, China.; 4 Electronic Materials Research Laboratory, Key Laboratory of the Ministry of Education and International Center for Dielectric Research, School of Electronic Science and Engineering, Xi’an Jiaotong University, Xi’an, Shaanxi 710049, China.; 5 Department of Chemistry, City University of Hong Kong, Kowloon, Hong Kong 999077, China.; 6 Department of Materials Science and Engineering, City University of Hong Kong, Kowloon, Hong Kong 999077, China.; 7 Department of Materials Science and Engineering, University of Washington, Seattle, WA 98195, USA.

## Abstract

Deep traps originated from the defects formed at the surfaces and grain boundaries of the perovskite absorbers during their lattice assembly are the main reasons that cause nonradiative recombination and material degradation, which notably affect efficiency and stability of perovskite solar cells (PSCs). Here, we demonstrate the substantially improved PSC performance by capping the photoactive layer with low-dimensional (LD) perovskitoids. The undercoordinated Pb ions and metallic Pb at the surfaces of the three-dimensional (3D) perovskite are effectively passivated via the Pb-I bonding from the favorably lattice-matched 3D/LD interface. The good stability and hydrophobicity of the LD (0D and 1D) perovskitoids allow excellent protection of the 3D active layer under severe environmental conditions. The PSC exhibits a power conversion efficiency of 24.18%, reproduced in an accredited independent photovoltaic testing laboratory. The unencapsulated device maintains 90% of its initial efficiency after 800 hours of continuous illumination under maximum power point operating conditions.

## Teaser

Low-dimensional perovskitoids cap the 3D photoactive perovskite layer to improve perovskite solar cell performance.

## INTRODUCTION

Organic-inorganic hybrid perovskite solar cells (PSCs) have recently made great progress in reaching a certified power conversion efficiency (PCE) of 25.5% because of the collective efforts in composition engineering, crystallization control, and defect management of perovskite materials (*1*–*5*). To achieve highly efficient solar cells, it is critical to control low trap-assisted [Shockley-Read-Hall (SRH)] nonradiative recombination rate. Traps in PSCs often originate from the defects formed at the surfaces and grain boundaries of the three-dimensional (3D) perovskite active layer. Specifically, iodide vacancies are commonly observed shallow traps, which can migrate to the interface under the bias of the electric field to affect device performance (*6*–*10*). Deep traps such as undercoordinated Pb^2+^ ions and metallic leads are usually found at the surfaces and grain boundaries of perovskite crystals due to tension created during the surface formation and improper processing conditions (*11*, *12*). These deep traps are particularly harmful, leading to severe SRH recombination to impede interfacial charge extraction and lower open-circuit voltage (*V*_oc_) of PSCs.

One very effective method to passivate defects in perovskite films is to use Lewis acids or bases because of their capability in accepting or donating electrons (*13*–*19*). However, the overall effectiveness of passivation and lasting stability is limited because Lewis acid or base ligands are distributed over the perovskite surface in a quasi-random manner due to relatively weak van der Waals interactions. Another widely used strategy is to use alkylammonium halide salts, which can be used to synthesize photoactive 2D perovskites with PbI_2_. Therefore, they can bound strongly with the 3D perovskite surface, forming an ultrathin (quasi-)2D “sheet” to passivate both cation and anion defects (*20*–*25*). However, the results from using this approach are not completely satisfactory due to the tendency of forming multicomponent structures in the quasi-2D phase and creating lattice strain during the formation of low-dimensional (LD) structures. Recently, perovskitoid has been proposed to describe the structures where the PbI_6_ octahedra are linked by edge and face sharing or combinations of corner, edge, and face sharing (*26*–*32*). Perovskitoids have the potential to effectively modify 3D perovskite due to its diverse PbI_6_ connection styles and high stability.

Hence, its partial success has motivated us to develop a more effective methodology to circumvent the abovementioned disadvantages. To this end, we have introduced both 1D and 0D perovskitoids as capping layer materials for 3D perovskite. The 1D and 0D perovskitoids, formulated as [*p*-(C_8_H_14_N_2_)]Pb_2_I_6_ or (*p*-PBA)Pb_2_I_6_ and [*m*-(C_8_H_14_N_2_)]_2_PbI_6_ or (*m*-PBA)_2_PbI_6_, are based on 1,4-bis(aminomethyl)benzene dihydroiodide (*p*-PBAI_2_) and 1,3-bis(aminomethyl)benzene dihydroiodide (*m*-PBAI_2_), respectively. Both 1D and 0D perovskitoids have intrinsically low defect densities and can withstand relatively high lattice strains; thus, they can serve as blocking channels for undesired SRH recombination and material degradation. In addition, the extended growth of uniform 1D and 0D coatings on 3D perovskite ensures effective and all-around passivation of the perovskite surfaces and grain boundaries. As a result, the best PCEs of 3D/1D- and 3D/0D-based devices can reach 23.84 and 24.49%, respectively, showing remarkable improvements over the 22.50% PCE achieved for a pure 3D-based device. A high PCE of 24.18% was validated by an independent laboratory for certification. The *V*_oc_ loss of the champion 3D/0D-based PSC is only 0.367 mV (from the 1.53-eV bandgap of the 3D perovskite). A significantly improved PSC stability can be achieved due to the higher humidity resistance of 1D and 0D capping materials.

## RESULTS

### Characterization of perovskitoid structure and 3D/LD films

**Fig. 1.**
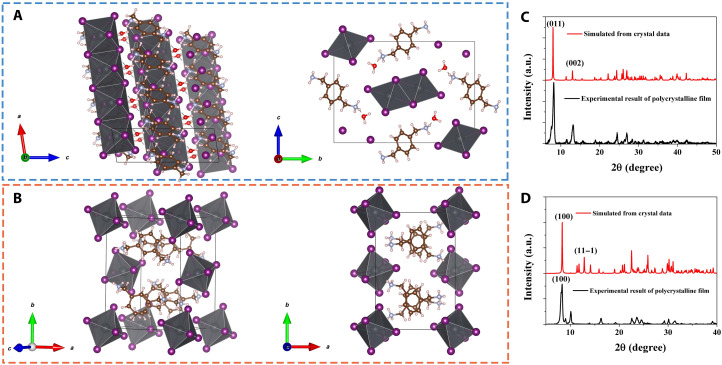
Structure of LD perovskitoids. (**A** and **B**) Atomic structure of (A) (*p*-PBA)Pb_2_I_6_ (1D) and (B) (*m*-PBA)_2_PbI_6_ (0D). (**C** and **D**) Polycrystalline film XRD (black) and simulated XRD from single-crystal structure (red) of (C) 1D and (D) 0D. a.u., arbitrary units.

Single-crystal x-ray diffraction (XRD) was used to characterize both LD perovskitoids based on *p*-PBAI_2_ and *m*-PBAI_2_. Both (*p*-PBA)Pb_2_I_6_ (Fig. 1A) and (*m*-PBA)_2_PbI_6_ (Fig. 1B) have a monoclinic lattice structure (space group *P*2_1_/c), with lattice constants *a* = 4.567 Å, *b* = 18.950 Å, *c* = 13.783 Å, and β = 98.796° for 1D and *a* = 10.952 Å, *b* = 15.539 Å, *c* = 8.865 Å, and β = 104.525° for 0D. In (*p*-PBA)Pb_2_I_6_, PbI_6_ octahedra are linked with each other via edge sharing, forming a series of 1D chains that are regularly arranged over the other two dimensions in space, and organic bivalent *p*-PBA cations are distributed among the inorganic, negatively charged 1D chains. Note that *p*-PBAI_2_–based perovskites that appeared in different structures vary with the precursor composition (fig. S1), similar with the guanidinium– and protonated thiourea–based perovskites (*33*, *34*). Differently, PbI_6_ octahedra in (*m*-PBA)_2_PbI_6_ are completely detached from each other so that each isolated octahedron is surrounded by several *m*-PBA cations and vice versa. The different assembling patterns of PbI_6_ octahedra are derived from para- and meta-substituted xylylenediammonium (PBA) isomers that can have hydrogen bonding formed between ammonium group and iodides to stabilize both perovskitoid structures. Polycrystalline film XRD spectra of pure 1D (Fig. 1C) and 0D (Fig. 1D) perovskitoid films prepared by a one-step method [evidenced by the ultraviolet–visible (UV-vis) absorption spectra in fig. S2] were also measured. They agree well with the simulated results based on the single-crystal data and the powder XRD results (figs. S3 and S4).

**Fig. 2.**
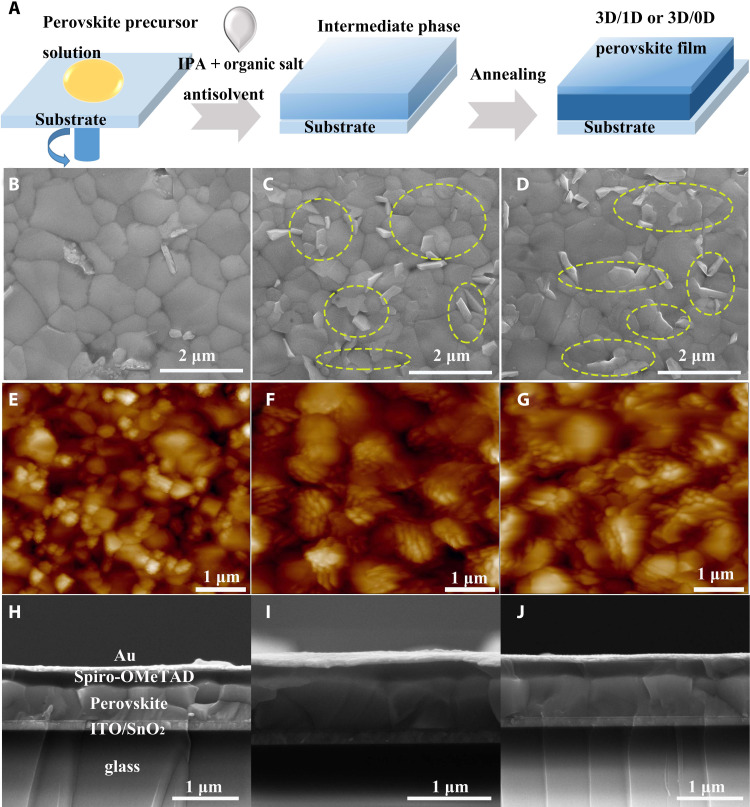
Fabrication and morphology of 3D/LD heterojunction perovskite films. (**A**) Scheme of one-step fabrication. Top surface SEM images (**B** to **D**), AFM images (**E** to **G**), and cross-sectional SEM images (**H** to **J**) of perovskite films fabricated with IPA, IPA + *p*-PBAI_2_, and IPA + *m*-PBAI_2_. Yellow dashed circles in (C) and (D) highlight the plate-like morphology.

In this work, the 3D/LD heterojunction films were fabricated using a one-step method (sketched in Fig. 2A), in which *p*- or *m*-PBAI_2_ salts were dissolved in the isopropyl alcohol (IPA) antisolvent and the divalent cations were assembled at the surface of the 3D perovskite. The morphology of perovskite films without and with the addition of PBAI_2_ was analyzed using scanning electron microscopy (SEM) and atomic force microscopy (AFM). Figure 2 (C and D versus B, and F and G versus E) shows a sharp contrast between the binary 3D/LD films and the pure 3D perovskite film. Obvious plate-like morphology with characteristic size of several hundred nanometers is observed at the surface and boundaries of the binary films (highlighted by yellow dashed circles in Fig. 2, C and D). This feature indicates that the growth of 3D perovskite crystals in the film was influenced by the formation of LD perovskitoids. The cross-sectional SEM images of devices (Fig. 2, H to J) show that the thickness of each perovskite film is around 650 nm, which can ensure the sufficient absorption of light.

### Lattice match between 3D perovskite and LD perovskitoids

**Fig. 3.**
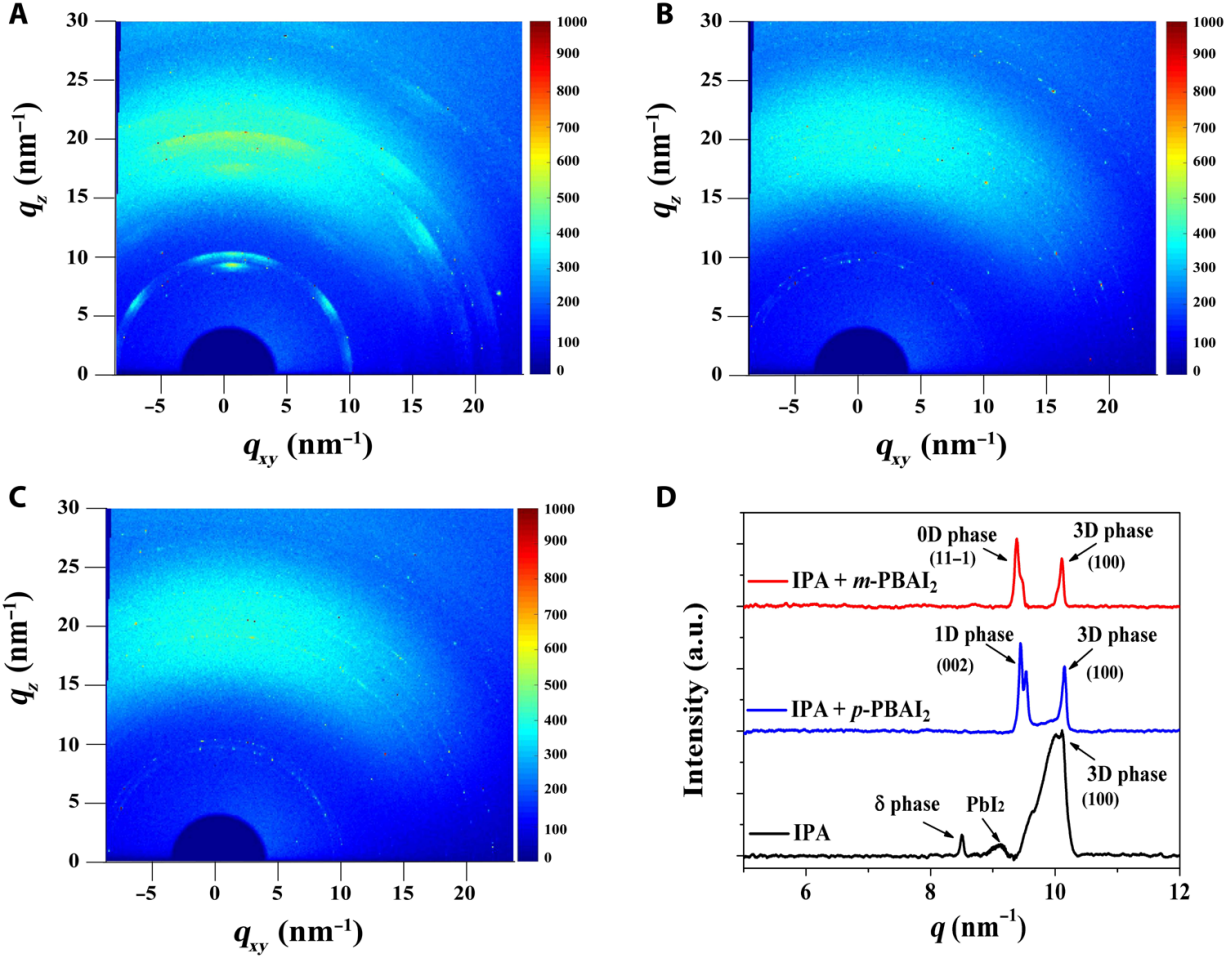
Structure characteristics of 3D/LD films. (**A** to **C**) GIWAXS patterns of perovskite films prepared with (A) IPA, (B) IPA + *p*-PBAI_2_, and (C) IPA + *m*-PBAI_2_. The x-ray beam at an incident angle of 0.1°. (**D**) Corresponding radially integrated intensity of GIWAXS data.

Grazing-incidence wide-angle x-ray scattering (GIWAXS) measurements of 3D and 3D/LD films were carried out to analyze the crystal orientations in the films. This technique also allows the exploration of local crystal phase distribution as a function of film depth by varying the grazing-incidence x-ray angle (fig. S5). Specifically, angles 0.1°, 0.2°, and 0.4°, at which we performed measurements, correspond to film depths (from the sample’s surface) of 3 to 5 nm, 10 to 20 nm, and 100 to 200 nm, respectively (*35*, *36*). GIWAXS patterns of the control (i.e., 3D), 3D/1D, and 3D/0D films are given in Fig. 3 (A to C, respectively), and the radially integrated intensities of GIWAXS data are summarized in Fig. 3D.

For the pure 3D perovskite film, three peaks at *q* ~10, ~8.5, and ~9.1 nm^−1^ are detected, belonging to the 3D perovskite phase, nonperovskite δ phase, and PbI_2_, respectively. Figure S6 further shows that the δ phase is only observed with the incident angle of 0.1°, i.e., surface sensitive. For the 3D/1D sample, a peak is observed at ~9.5 nm^−1^, corresponding to the 1D perovskitoid phase, and similarly, a peak at ~9.3 nm^−1^ emerges from the 3D/0D film, indicating the 0D perovskitoid phase. In both 3D/LD samples, the nonperovskite δ phase is substantially reduced, signifying that the LD perovskitoids can restrain the formation of the photoinactive nonperovskite phase (*37*).

The phase distribution analysis at different depths (figs. S6 to S9) shows that the LD phase peak in both 3D/LD films disappears when the incident angle increases from 0.1° to 0.2° and further. Therefore, we can conclude that the LD perovskitoid structure is coated at the surface of the 3D perovskite with an overall thickness of ~5 nm. As a result of the ultrathin LD coating, the PbI_2_ peak is not observed at the incident angle 0.1° for both 3D/LD films but is noticeable in deeper regions (incident angles 0.2° and 0.4°). Hence, the excess PbI_2_ in the bulk of the 3D film or even close to the surface is not influenced. XRD measurements of both binary 3D/LD films also demonstrate the existence of excess PbI_2_ (fig. S10).

**Fig. 4.**
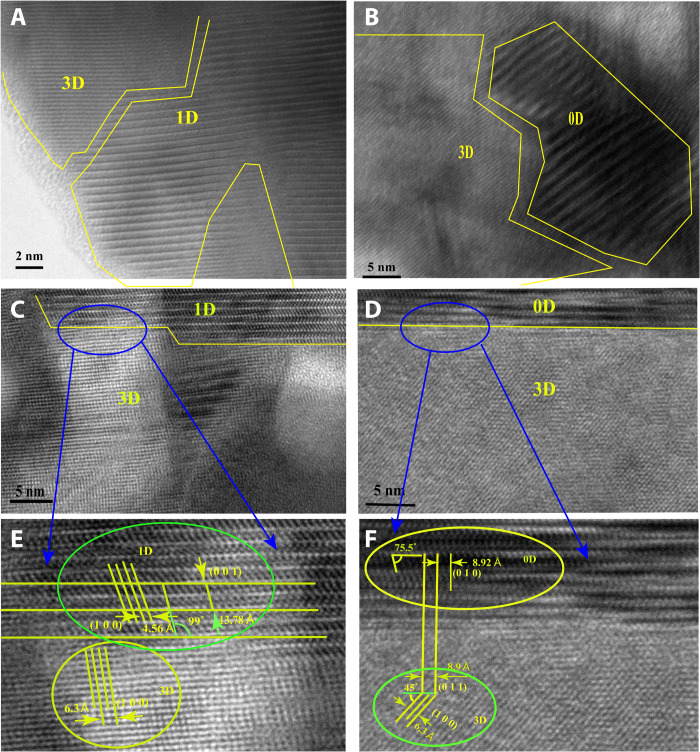
Lattice matching between 3D perovskite and LD perovskitoids. (**A** and **B**) Powder HRTEM of (A) 3D/1D BHJ and (B) 3D/0D BHJs. (**C** and **D**) Cross-sectional TEM of (C) 3D/1D film and (D) 3D/0D films. (**E** and **F**) Lattice-matching analysis based on highlights of blue circled regions in (C) and (D), respectively.

To further analyze the formation of 3D/LD interfaces, both bulk heterojunctions (BHJs; denoted by 3D@LD) and planar 3D/LD films were characterized by high-resolution transmission electron microscopy (HRTEM). The 3D@LD BHJ films were prepared from mixed precursor solutions with a molar ratio 3D:LD = 7:3. Powder HRTEM images of 3D@1D and 3D@0D BHJs (Fig. 4, A and B, respectively) show that both 3D perovskite and LD perovskitoids were successfully formed and closely linked with each other. Proper lattice matching is observed at the 3D-LD border regions.

**Fig. 5.**
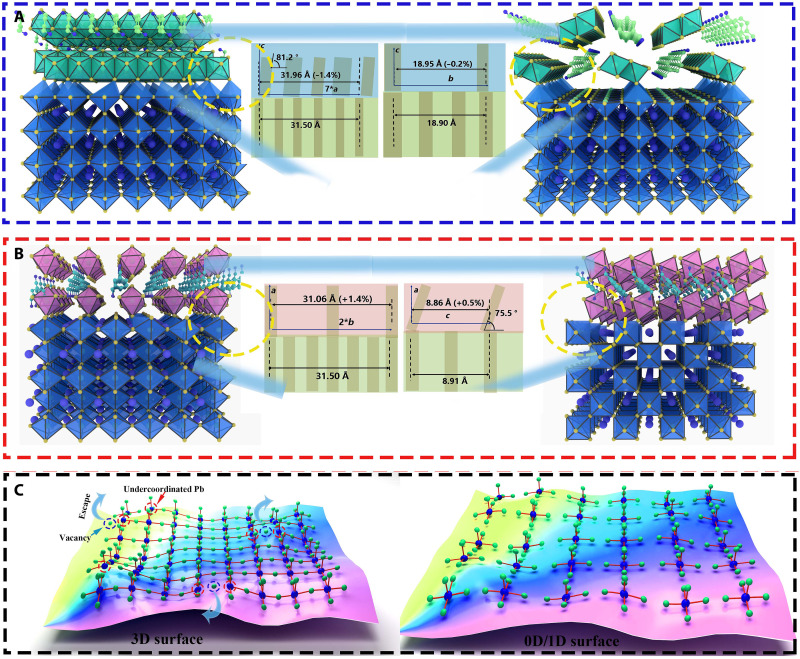
Schemes of lattice matching. (**A**) 3D/1D. (**B**) 3D/0D. (**C**) Scheme of 1D and 0D passivation.

For planar heterojunction 3D/1D and 3D/0D thin films, we performed cross-sectional HRTEM measurements (Fig. 4, C to F) on samples that were deposited on indium tin oxide (ITO) using the method sketched in Fig. 2A, coated with a protective platinum layer, and further processed by focused ion beam (FIB) cutting. The images show that both 1D and 0D perovskitoids were successfully formed at the surface of the 3D perovskite with proper lattice matching. On the basis of the cross-sectional HRTEM images and analysis of lattice constants, we can derive the lattice-matching schemes of both 3D/1D and 3D/0D as shown in Fig. 5 and fig. S11.

Specifically, the interface in 3D/1D is formed between the (001) surface of the 3D perovskite and the (001) surface of the 1D perovskitoid, where the [100] and [010] lattice vectors (both slightly compressed) of 1D are aligned with the [001] and [100] lattice vectors of 3D, respectively. The 3D/0D interface is formed between the 3D-(011) surface and the 0D-(100) surface, where the [001] and [010] lattice vectors (both slightly elongated) of 0D are aligned with the [100] and [0-11] lattice vectors of 3D, respectively. The matching between the 3D and the LD lattices has the following characteristics. First, it does not cause significant strain on the LD capping layer materials (the largest strain is 1% for the 0D-[010] lattice vector, which is readily tolerable). Second, the Pb-I coordinating bonds are regularly formed at the interface to stabilize the overall binary complex and create charge transport channels. Third, the density of interfacial Pb-I bonds is moderate so that they do not cause strong lattice distortion in both the LD capping material and the 3D substrate.

### Device architecture and photovoltaic performance

**Fig. 6.**
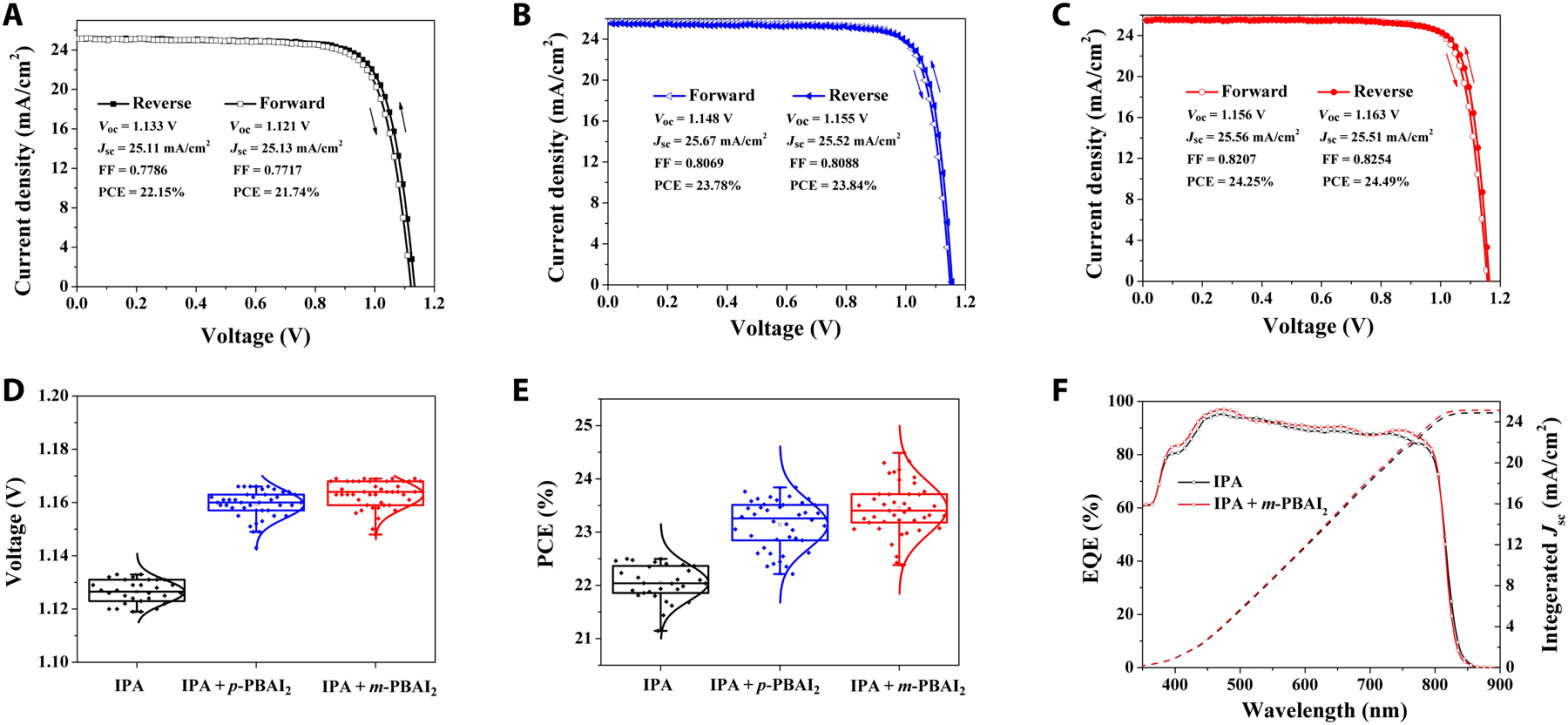
Performance of PSCs. (**A** to **C**) Typical *J-V* curves of (A) pure 3D, (B) 3D/1D, and (C) 3D/0D devices. FF, fill factor. (**D** and **E**) Statistics of (D) *V*_oc_ and (E) PCE of devices prepared with the optimum concentration and without organic salts. (**F**) Photon-to-electron conversion efficiency curves for pure 3D (IPA) and 3D/0D (IPA + *m*-PBAI_2_) devices. EQE, external quantum efficiency.

The photovoltaic performance of PSCs was explored on the basis of the typical planar device structure ITO/SnO_2_/perovskite/2,2’,7,7’-tetrakis-[*N*,*N*-di(4-methoxyphenyl)amino]-9,9’-spirobifluorene (spiro-OMeTAD)/Au. The concentration of the IPA solution of organic salts was optimized to 0.1 mg/ml for both *p*- and *m*-PBAI_2_ (data summarized in tables S1 to S6). The PCE of PSCs will decrease if the concentrations of PBAI_2_ are further increased, possibly due to too thick LD coatings with relatively low conductivity. Figure 6 (A to C) shows the typical *J-V* curves of 3D, 3D/1D, and 3D/0D devices. The statistics of *V*_oc_ and PCE of both pure 3D and 3D/LD devices are presented in Fig. 6 (D and E, respectively). Integrated photocurrents calculated from the measured external quantum efficiencies of both pure 3D and 3D/0D devices (Fig. 6F) are 24.87 and 25.10 mA cm^−2^, respectively, both agreeing well with the short-circuit current (*J*_sc_) derived from the *J-V* measurements.

The best PCEs of pure 3D, 3D/1D, and 3D/0D devices are 22.49, 23.84, and 24.49%, respectively. The traditional 3D/2D device was also fabricated with phenethylammonium iodide (PEAI), and a PCE of 23.15% was obtained (fig. S12). Steady-state photocurrent at the maximum power point of the 3D/0D device is shown in fig. S13. The hysteresis index is remarkably decreased from 1.85% (for the pure 3D device) to 0.25% for 3D/1D and 0.98% for 3D/0D PSCs upon the introduction of LD perovskitoids (table S1). Such a significantly reduced *J*-*V* hysteresis should be the combined results of better energy-level alignment, suppressed ion migration, and surface passivation (*13*, *38*–*40*).

The best device performance was validated by an accredited independent photovoltaic testing laboratory, reporting a PCE of 24.18% (with *J*_sc_ = 25.96 mA/cm^2^, *V*_oc_ = 1.151 V, and fill factor = 80.91%) for the 3D/0D-based PSC. The test report is shown in fig. S14. Moreover, a highly flexible 3D/0D-based device was also prepared, with its *J-V* curves and photovoltaic performance parameters presented in fig. S15 and table S7. It yields a PCE as high as 21.71%. A 3D/0D device with an active area of 1 cm^2^ was also fabricated with a PCE of 22.94% (fig. S16).

### Passivation of surface defects

**Fig. 7.**
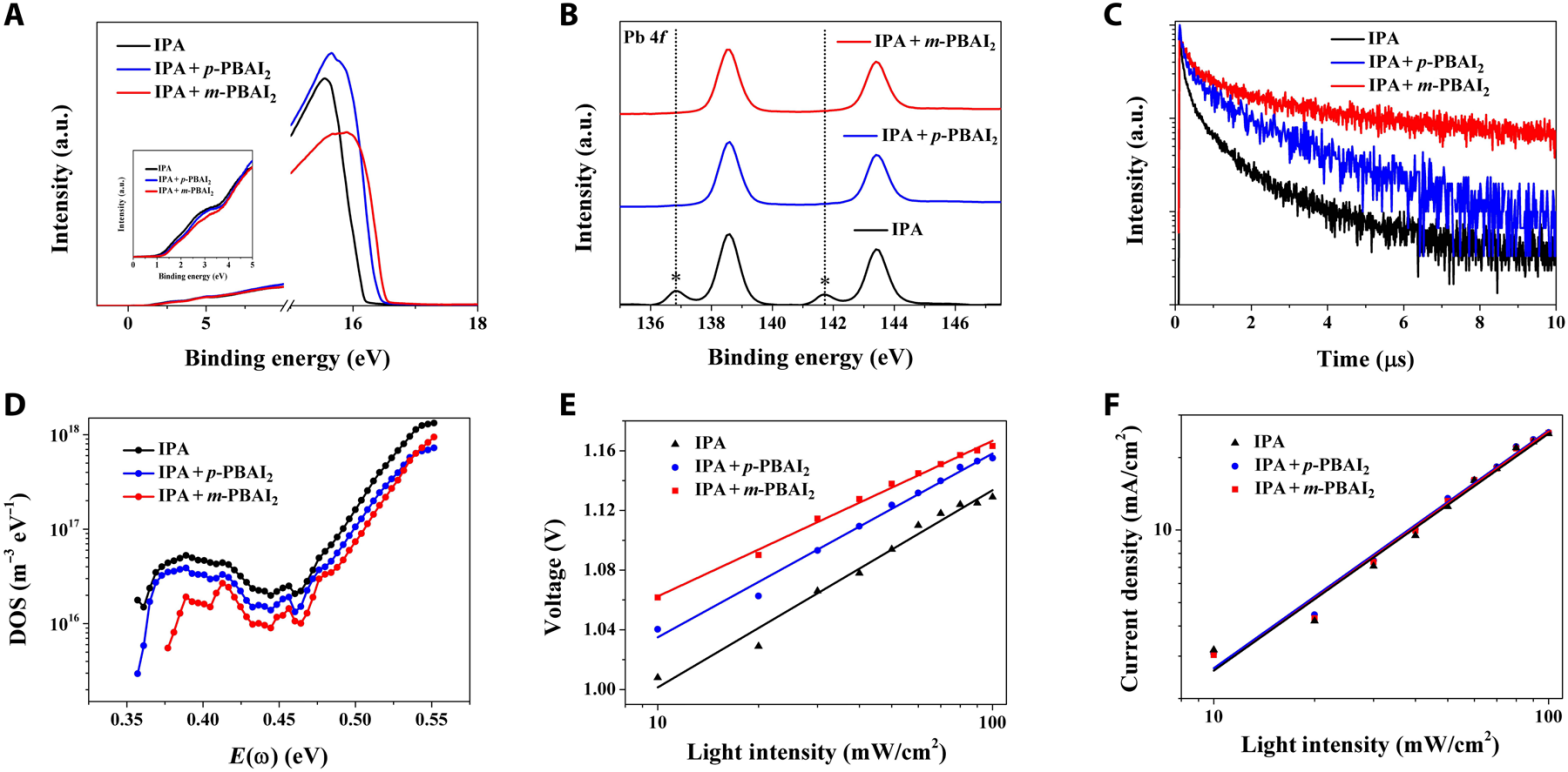
Characterization of surface defect passivation. (**A**) UPS spectra measured with the onset of bias voltage versus gold. (**B**) XPS spectra of Pb 4*f*. (**C**) TRPL spectra (with samples deposited on quartz) for the films. (**D**) Trap density of states (DOS) from TAS. (**E** and **F**) Light intensity dependence of (E) *V*_oc_ and (F) *J*_sc_ for investigated PSC devices.

To investigate the impact of LD perovskitoid capping layer on the electronic structure of the 3D perovskite film and eventually the device performance, ultraviolet photoemission spectroscopy (UPS), x-ray photoelectron spectroscopy (XPS), steady-state photoluminescence (PL) spectrum, time-resolved PL (TRPL), and thermal admittance spectroscopy (TAS) of pure 3D and 3D/LD films were measured. From the UPS measurements (Fig. 7A), the valence band maximum is estimated to be −6.13, −6.00, and −5.92 eV for the pure 3D, 3D/1D, and 3D/0D films, respectively. On the basis of the bandgap of perovskite (estimated in fig. S17), the energy band diagrams of the bare perovskite and the perovskite/hole-transporting layer (HTL) junction without and with the LD perovskitoid capping layer can be sketched as shown in fig. S18. These diagrams rationalize the application of wide bandgap LD perovskitoids as a capping layer efficiently inhibiting the undesired electron transfer from the photoactive 3D perovskite to HTL, thus preventing charge carrier quenching and SRH recombination.

XPS measurements (Fig. 7B and fig. S19) were carried out to study the chemical states of elements (more specifically Pb) at the surfaces of different films. Figure 7B shows that the pure 3D perovskite film exhibits two major peaks (138.6 eV for 4*f*_7/2_ and 143.5 eV for 4*f*_5/2_) corresponding to the Pb^2+^ species with saturated coordination and small peaks at lower binding energies (136.8 eV for 4*f*_7/2_ and 141.7 eV for 4*f*_5/2_) that are attributed to the undercoordinated metallic Pb (Pb^0^) (*41*–*45*). The introduction of LD capping layers can effectively passivate these surface Pb-related defects and fix the surface dangling bonds as evidenced by the disappearance of Pb^0^ peaks in both 3D/1D and 3D/0D perovskites.

TRPL curves (Fig. 7C) can be used to analyze the dynamics of photoexcited charge carriers by fitting the decay curves using a biexponential function as

$$Y=A_{1}\text{exp}(-\frac{t}{\tau_{1}})+A_{2}\text{exp}(-\frac{t}{\tau_{2}})+y_{0}$$

with τ_1_ and τ_2_ representing the fast and slow decay time constants that are associated with defect-induced nonradiative and radiative recombination, respectively (*46*). The fitting results (table S8) of τ_2_ for the pure 3D, 3D/1D, and 3D/0D films are 0.82, 1.13, and 1.34 μs, respectively. The elongated slow decay time constant indicates the reduced defect density due to passivation by the capping layer. Besides, fig. S20 shows that the pure 3D film exhibits similar steady-state PL intensities excited from either side, while the intensity at the 3D perovskite side is obviously stronger than the glass side for both 3D/LD films. This contrast provides a compelling evidence that the imperfections were minimized by the LD coating. Trap density of states measured with TAS (Fig. 7D) shows an obvious trend of decreasing densities of both shallow (0.35 to 0.4 eV) and deep trap states (above 0.4 eV) along the control and 3D/1D- and 3D/0D-based devices (*42*, *47*). The same conclusion can be inferred from the *J-V* curves of the hole-only device under dark condition (fig. S21).

Light intensity–dependent *J-V* measurements were carried out to investigate the influence of LD perovskitoid capping layer on charge extraction and recombination kinetics. Figure 7E illustrates the dependence of *V*_oc_ on the incident light intensity, which can be fitted according to the equation of *V*_oc_ = ε*kT* ln (*I*)/*e* + const, where ε is the ideality factor, *k* is the Boltzmann constant, *e* is the elementary charge, and *T* is the temperature (*48*). From the fitting results, we observe that the diode ideality factors are 1.72, 1.53, and 1.41 for the pure 3D-, 3D/1D-, and 3D/0D-based PSCs, respectively. Since a smaller ideality factor corresponds to lower trap-assisted monomolecular SRH recombination, this measurement further supports the lower trap densities in the capped perovskite films (*49*). In addition, Fig. 7F presents the dependence of *J*_sc_ on the incident light intensity (both in logarithmic scales) fitted according to *J*_sc_ ∝ α*I*. The slopes of all three types of devices are nearly identical to each other, indicating that all of them exhibit efficient charge carrier extraction (*50*, *51*). Moreover, the results of space charge–limited current measurement also demonstrates that the ultrathin coating of LD framework does not inhibit charge transport and extraction (fig. S21). To summarize, results of all measurements support that LD (especially 0D) perovskitoid capping layer can efficiently passivate the defects at the surfaces and grain boundaries of the 3D perovskite film and thus significantly improve the device efficiency.

### Stability of PSCs

Last, both LD perovskitoid materials are found to be more resistant to water and oxygen than 3D perovskites, which can serve as a protecting “wall” for 3D perovskite-based devices. The LD perovskitoid materials show excellent damp and heat stability (fig. S22). Figure S23 shows that the surface hydrophobicity of samples with either LD capping layer is higher than that of the pure 3D perovskite film, as evidenced by the increased water contact angle from 52.91° to 67.05° and 76.03° for the pure 3D, 3D/1D, and 3D/0D films, respectively. Time-dependent contact angle measurements show that both 3D/1D and 3D/0D films exhibit better moisture resistance. Therefore, we can conclude that the capping layer can effectively prevent water from diffusing into the perovskite film to enhance the stability of 3D/LD films and devices in humid environment (fig. S24). Figure S25 shows that both 3D/LD PSCs can maintain 90% of their initial efficiencies after being stored in ambient air for 2000 hours, while the efficiency of pure 3D PSC drops to 80%. Damp and heat stress tests have also been carried out for unencapsulated PSCs under the condition of 85°C and 85% relative humidity. The efficiency of the pure 3D PSC drops markedly to 55% of its initial value, while both 3D/LD PSCs retain 80% of their initial efficiencies after being exposed under these conditions for 150 hours (fig. S25B). Moreover, the operational stability of unencapsulated devices under 1-sun light-emitting diode (LED) irradiation with maximum power point operation (MPP) tracking in air was investigated (fig. S25C). It revealed that the 3D/0D device only shows 10% PCE loss after 800 hours of aging, whereas a PCE loss in control device is up to 40%. The significant improvement of stability in 3D/LD PSC is attributed to better moisture resistance of LD capping layers, reduced trap density of 3D perovskite surface, and tight bonding of the 3D/LD interface.

## DISCUSSION

In summary, an innovative passivation strategy is developed by coating 1D or 0D perovskitoid capping layer on the 3D perovskite. This process markedly improves both photovoltaic efficiency and long-term stability of the devices. Both LD perovskitoids can form extended interfaces with the 3D perovskite to stabilize the complex. It results in significantly reduced SRH recombination and enhanced moisture resistance. Consequently, the PCE of the champion 3D/0D-based PSC can reach as high as 24.49% (24.18% certified). In addition, a flexible 3D/0D-based device with a high PCE of 21.76% can also be fabricated to demonstrate the general applicability of this novel approach to different forms of devices. This study establishes a new material engineering scheme for PSC with enormous numbers of 1D and 0D perovskitoid candidates that can be used to further improve the PSC performance and stability.

## MATERIALS AND METHODS

### Materials

*N*,*N*′-dimethylformamide (DMF), dimethyl sulfoxide (DMSO), IPA, and SnO_2_ colloid precursor are from Alfa Aesar. Spiro-OMeTAD, acetonitrile (ACN), bis(trifluoromethanesulfonyl)imide lithium (Li-TFSI), *tert*-butylpyridine (tBP), CsI, *p*-PBA, and *m*-PBA were purchased from Sigma-Aldrich. PbI_2_ was purchased from Tokyo Chemical Industry. 3-Phenyl-2-propenammonium iodine (PPEAI), methylammonium chloride (MACl), methylammonium bromide (MABr), and formamidinium iodide (FAI) were synthesized as previously reported (*52*). Tris(2-(1*H*-pyrazol-1-yl)-4-tertbutylpyridine)-cobalt(III) tris(bis(trifluoromethylsulfonyl)imide) (FK209) was purchased from Xi’an Polymer Light Technology Corp. Chlorobenzene (CB) was obtained from Arcros. All reagents were purchased from commercial vendors and used as received.

### Synthesis of organic materials

#### 1,4-Bis(aminomethyl)benzene iodine

*p*-PBAI_2_ (1 g) was added to EtOH (10 ml) in ice water bath and then added with 2 ml of hydriodic acid (HI, 57%, 1.1 equiv). The reaction mixture was stirred over 2 hours at ambient temperature. The mixture was concentrated under vacuum, poured into diethyl ether (10 ml), filtered, extensively washed with diethyl ether and IPA, and dried under vacuum to afford *p*-PBAI_2_ as a white solid.

#### 1,3-Bis(aminomethyl)benzene iodine

*m*-PBAI_2_ (1 ml) was added to EtOH (10 ml) in ice water bath and then added with 2 ml of HI (57%,1.1 equiv). The reaction mixture was stirred over 2 hours at ambient temperature. The mixture was concentrated under vacuum, poured into diethyl ether (10 ml), filtered, extensively washed with diethyl ether and IPA, and dried under vacuum to afford *m*-PBAI_2_ as a white solid.

### Growth of single crystals

#### p-PBAPb_2_I_6_ single crystals

PbI_2_ powder (0.922 g) was dissolved in 10 ml of HI (57%), 0.272 g of *p*-PBA was added to the solution, and then the solution was kept stirring at 100°C for 4 hours. To grow the *p*-PBAPb_2_I_6_ single crystal, the hot supernatant was filtered with a 0.22-μm filter and then decreased by 1°C per hour until it was 40°C to obtain a *p*-PBAPb_2_I_6_ single crystal.

#### m-PBA_2_PbI_6_ single crystals

PbI_2_ powder (0.922 g) and *m*-PBAI_2_ (0.78 g) were dissloved in 2 ml of DMF at 100°C, and then the amount of ethyl acetate was added to the solution until precipitation. Then, with a 0.22-μm filter, the saturated solution was kept in an ethyl acetate atmosphere at 100°C and then decreased by 1°C per hour until it was 70°C to obtain an *m*-PBA_2_PbI_6_ single crystal. The *m*-PBA_2_PbI_6_ single crystals can be obtained as the growth process of *p*-PBAPb_2_I_6_ single crystals.

### Single-crystal structure determination

Single-crystal XRD was executed on a SuperNova diffractometer with graphite-monochromatized Mo-Ka radiation (*l* = 0.71073 Å) at 150 K. Data procession was conducted using CrystalClear software. The structures were solved by direct methods and refined by the full-matrix least-squares techniques on F2 using the SHELXLTL-97 software package and olex2. The nonhydrogen atoms were refined anisotropically and located by the difference Fourier maps. The positions of the hydrogen atoms were fixed geometrically at the calculated distances and allowed to ride on the parent atoms. The supplementary crystallographic data of *m*-PBA_2_PbI_6_ were provided in Cambridge Crystallographic Data Centre (CCDC) 2088163. The supplementary crystallographic data of *p*-PBAPb_2_I_6_ were provided in CCDC 2088164.

### Device fabrication

A 0.5-mm-thick ITO (15 ohm/square) glass was cleaned by sequentially washing with detergent, deionized water, and IPA. Before using, the ITO was cleaned by ultraviolet ozone (UVO) for 15 min. Then, the substrate was spin-coated with SnO_2_ (3000 rpm, 30 s) and baked on a hot plate in ambient air at 180°C for 30 min. After cooling to room temperature, the substrate was treated by UVO for 15 min. Here, 1.6 M CsFAMA mixed perovskite precursor solution was prepared by mixing PbI_2_ (1.6 mM), FAI (1.392 mM), MABr (0.08 mM), and MACl (22 mg) in 1 ml of DMF-DMSO (4:1, v/v) mixture, and 47 μl of CsI (1.5 M in DMSO) was added and stirred for 3 hours. CsFAMA perovskite films were deposited by spin coating (2000, 10 s/6000, 25 s), and 150 μl of IPA or organic salt IPA solvents was used as antisolvent 10 s before the end of the spinning. Then, the films were annealed at 100°C for 10 min and then increased to 150°C for 15 min. After cooling to room temperature, PPEAI (7 mg/ml) was spin-coated on the film at 3000 rpm. 17.8 μl of Li-TFSI/ACN (520 mg/ml), 28.5 μl of tBP, and 20 μl of FK209/ACN (200 mg/ml) were added to 1 ml of Spiro-OMeTAD/CB solution (90 mg/ml). This solution was then coated at 3000 rpm for 30 s. Last, Au back electrode was deposited by thermal evaporation. The active area was 0.0706 cm^2^.

### Characterization

UPS and XPS measurements were performed with ESCALAB 250Xi (Thermo Fisher Scientific) (by using Al-Kg x-ray source) under high vacuum (10^−9^ is derived). For work function measurement with UPS, 10-V bias was applied and Au was used as a reference. UV measurement was completed with a UV-vis/near-infrared spectrometer (HITACHI U-3010, Japan). Samples for the HRTEM measurements were prepared by ultrasonic dispersion (in CB) of powders that had been scraped from the 3D@LD films. For the 3D/LD film, the samples were prepared using a Helios NanoLab 450 model FIB (FEI) and field-emission SEM. The FIB milling/polishing process ensured that the samples had a smooth surface. Furthermore, the FIB-based milling process allowed the sample to be thinned to 100 nm, as required for HRTEM characterization. HRTEM images were collected using a JEOL JEM-2100F instrument. GIWAXS measurements were performed at beamline BL14B1 of the Shanghai Synchrotron Radiation Facility. *J*-*V* measurements were carried out using a Keithley 2400 sourcemeter in ambient environment of 24°C and 30% relative humidity. Illumination was provided by an XES-301S (SAN-EI) solar simulator with AM1.5G spectrum, and light intensity of 100 mW/cm^2^ was calibrated by means of a KG-5 Si diode. The devices were measured in both reverse scan (1.2 versus −0.05 V, step 0.001 V) and forward scan (−0.05 versus 1.2 V, step 0.001 V) with a scan rate of 0.06 V/s. The active area was determined by the aperture shade masks (0.04912 cm^2^) (certified by National Institute of Metrology, China, no. CDjc2019-0206). Devices were taken out for external quantum efficiency measurements using a solar cell system (Solar Cell Scan 100, Zolix), with the light intensity calibrated with a standard single-crystal Si photovoltaic cell. The XRD patterns were taken on Bruker ECO D8 series. PL and time-resolved PL spectra were carried out with a spectrofluorometer (FS5, Edinburgh Instruments), and 450-nm pulsed laser was used as an excitation source for the measurement.

## Supplementary Materials

### This PDF file includes:

Figs. S1 to S25 Tables S1 to S8 References
