# Supplementary material for: Highly efficient and stable perovskite solar cells enabled by low-dimensional perovskitoids
Source: Sci Adv. 2022 Jan 26;8(4):eabk2722. doi: 10.1126/sciadv.abk2722 (PMC8791463; doi:10.1126/sciadv.abk2722)
Supplement: Supplementary file 1 — Figs. S1 to S25 Tables S1 to S8 References [file sciadv.abk2722_sm.pdf]

Science Advances
AAAS

Supplementary Materials for

Highly efficient and stable perovskite solar cells enabled by low-dimensional perovskitoids

Jinbo Chen, Yingguo Yang, Hua Dong*, Jingrui Li*, Xinyi Zhu, Jie Xu, Fang Pan, Fang Yuan, Jinfei Dai, Bo Jiao, Xun Hou, Alex K.-Y. Jen*, Zhaoxin Wu*

*Corresponding author. Email: donghuaxjtu@xjtu.edu.cn (H.D.); jingrui.li@xjtu.edu.cn (J.L.); alexjen@cityu.edu.hk (A.K.-Y.J.); zhaoxinwu@xjtu.edu.cn (Z.W.)

Published 26 January 2022, *Sci. Adv.* **8**, eabk2722 (2022)
DOI: 10.1126/sciadv.abk2722

This PDF file includes:

Figs. S1 to S25
Tables S1 to S8
References

Other Supplementary Material for this manuscript includes the following:

Dataset S1 and S2

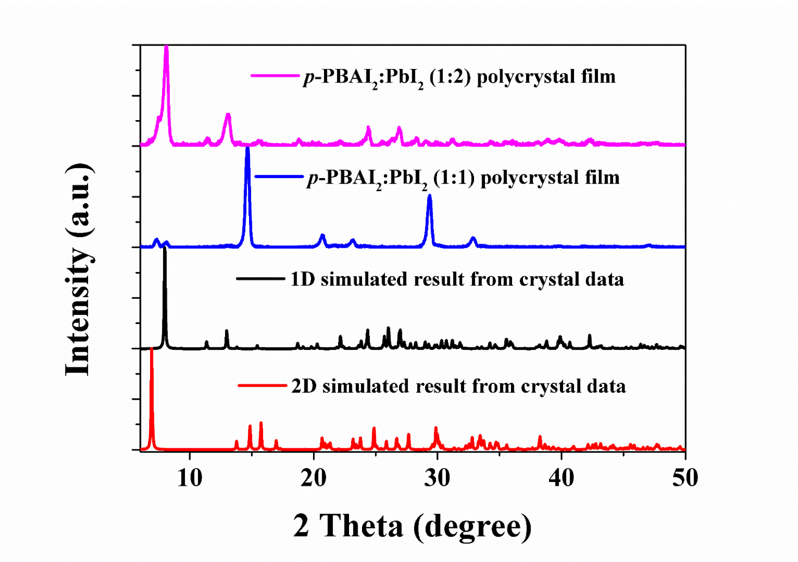

Fig. S1 XRD patterns of perovskite film with different composition. The XRD result of *p*-$PBAI_2$ based perovskite, (red line) the simulated result of 2D crystal data (*53*), (black line) the simulated result of 1D crystal data, (blue line) the XRD result of *p*-$PBAI_2$ based perovskite polycrystalline film fabricate with *p*-$PBAI_2$:PbI_2 (1:1) on glass, (magenta line) with *p*-$PBAI_2$:PbI_2 (1:2) on glass.

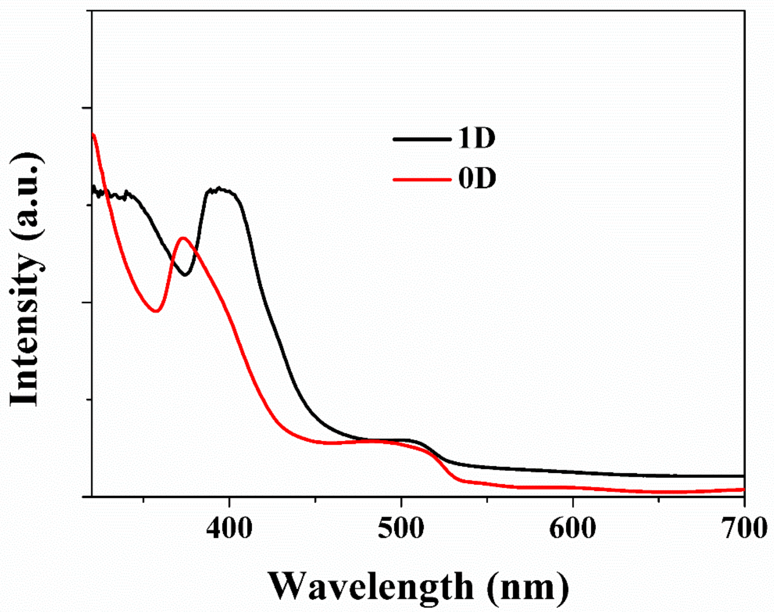

Fig. S2. UV-vis absorption spectra of perovskitoid film. UV-vis absorption spectra of pure 1D (black) and 0D (red) perovskitoid film.

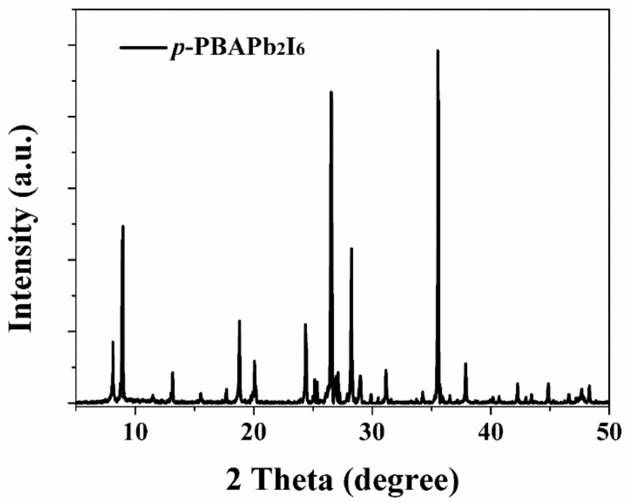

Fig. S3. Powder XRD of perovskitoid. The powder XRD of *p*-$PBAPb_2I_6$.

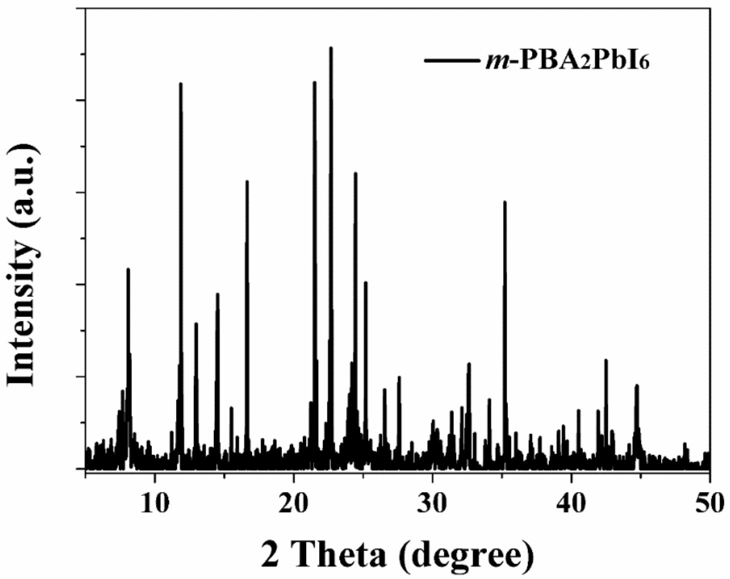

Fig. S4. Powder XRD of perovskitoid. The powder XRD of *m*-PBA_2PbI_6.

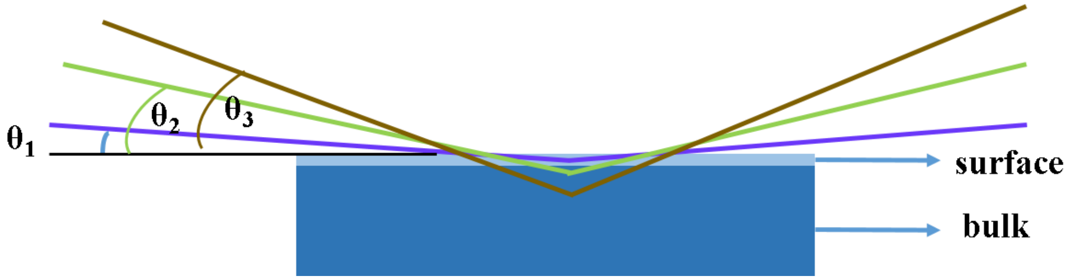

Fig. S5. Scheme of GIWAXS test. The scheme of GIWAXS with the incident beam incident on the sample at different angle.

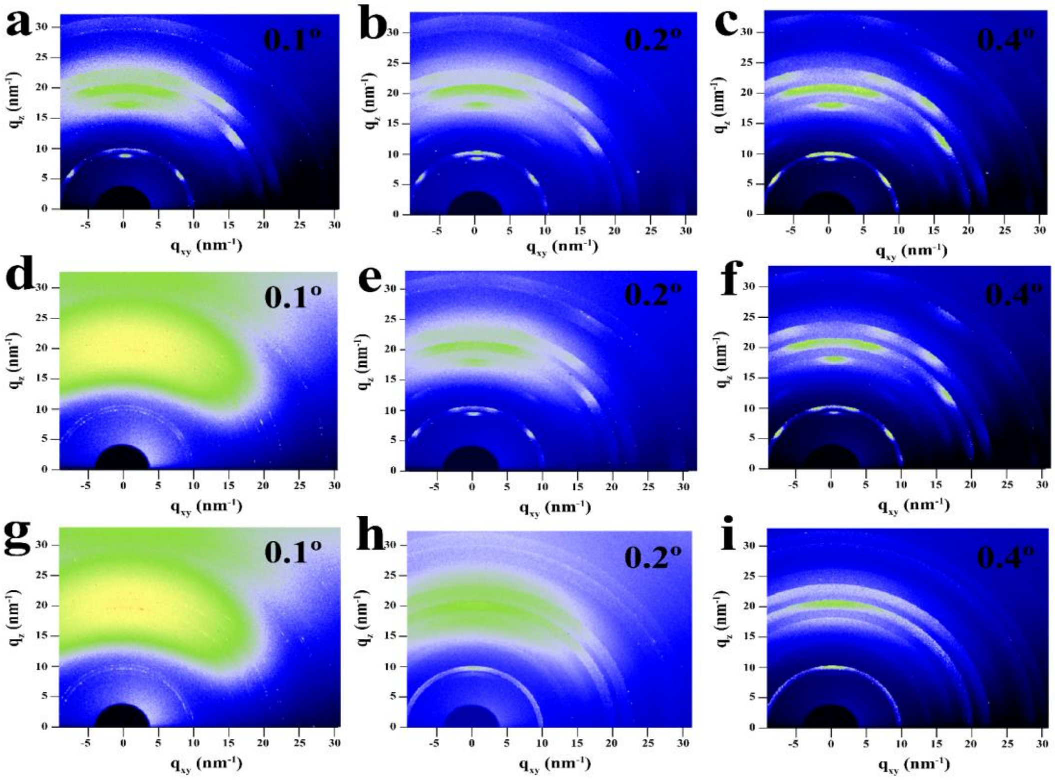

Fig. S6. The GIWAXS patterns of perovskite films with different incident angle. (a), (b), (c) pristine film. (d), (e), (f) 3D/1D film. (g), (h), (i) 3D/0D film.

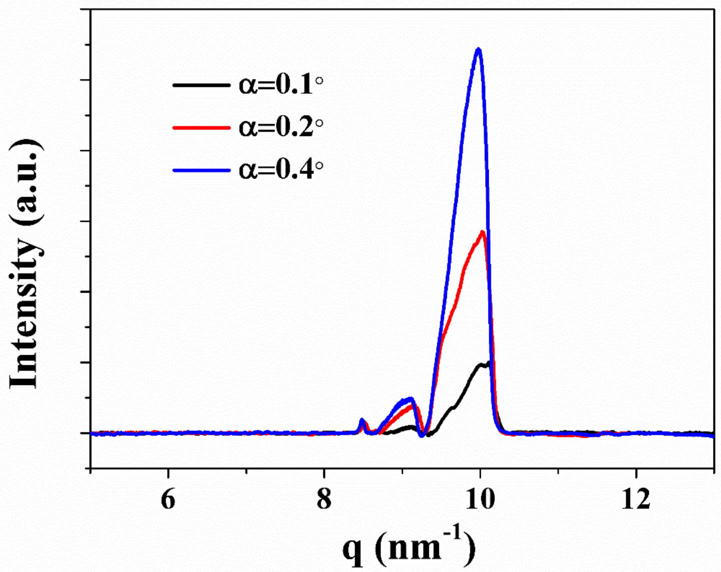

Fig. S7. Radially integrated intensity of GIWAXS data. The corresponding radially integrated intensity of pure 3D film GIWAXS data.

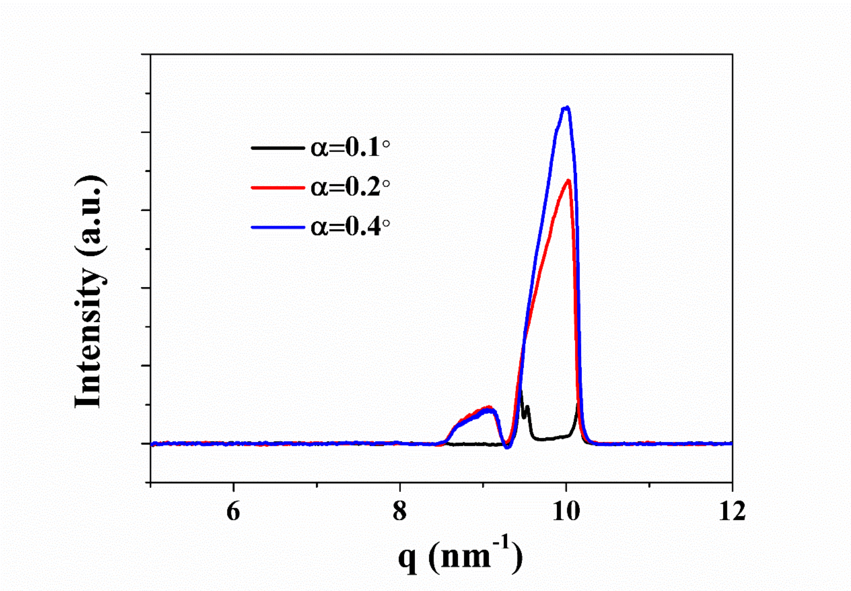

Fig. S8. Radially integrated intensity of GIWAXS data. The corresponding radially integrated intensity of 3D/1D film GIWAXS data.

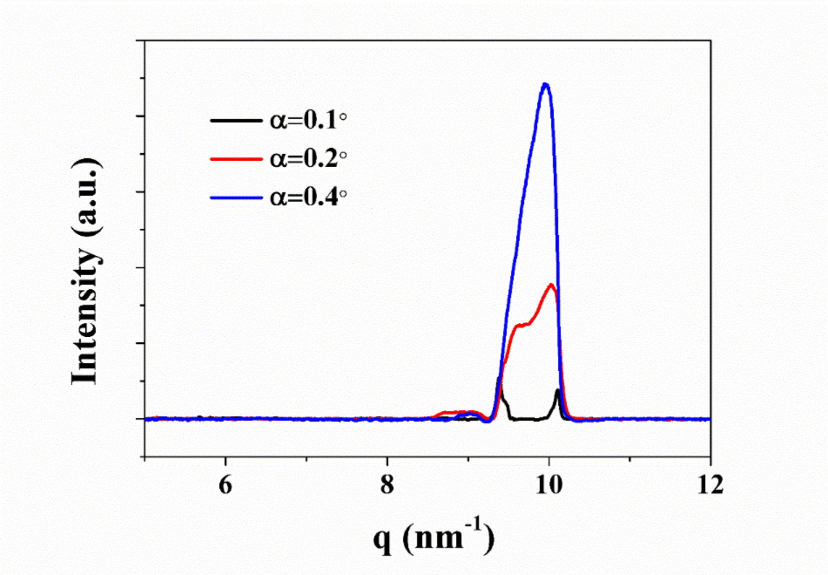

Fig. S9. Radially integrated intensity of GIWAXS data. The corresponding radially integrated intensity of 3D/0D film GIWAXS data.

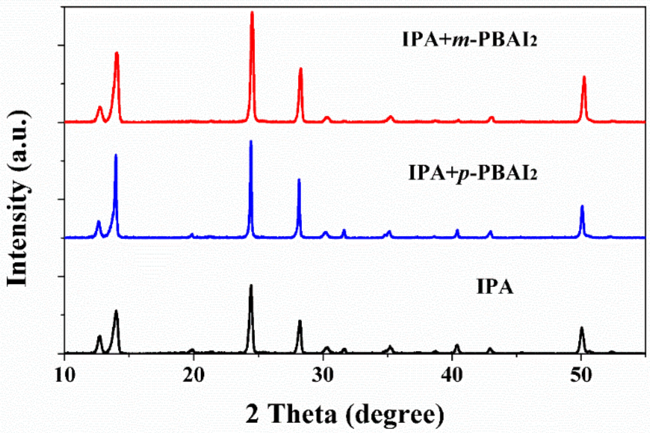

Fig. S10. XRD patterns of different perovskite films. The perovskite films were fabricated with IPA, IPA+*p*-$PBAI_2$, IPA+*m*-$PBAI_2$.

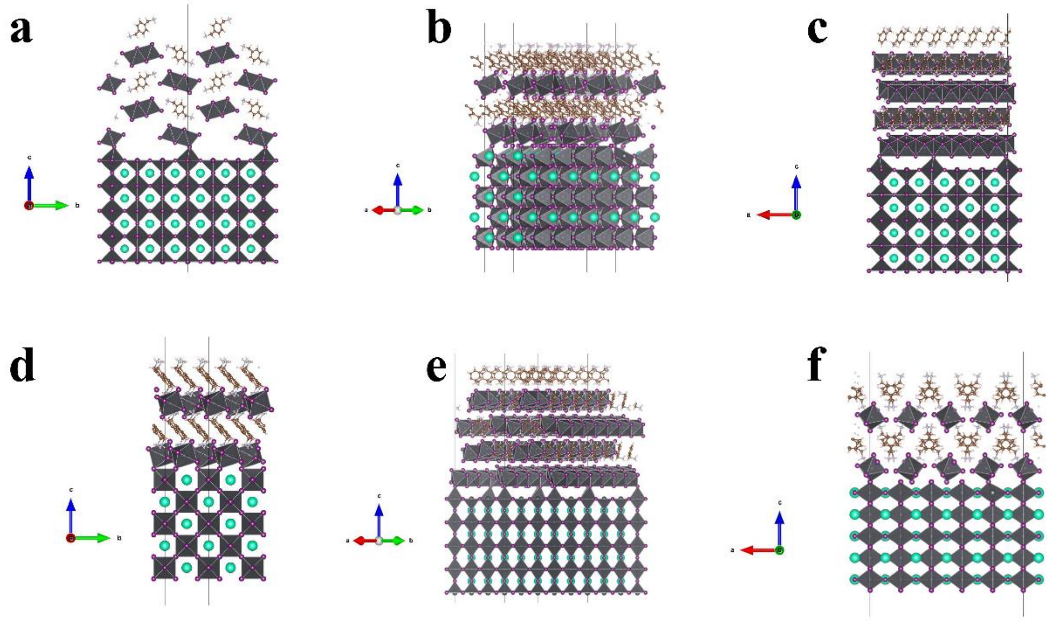

Fig. S11. Schemes of formation of the 3D/LD interfaces. (a)-(c) 3D/1D, and (d)-(f) 3D/0D. Shown are side-views from different directions. FA cations are symbolized in light green, while Pb and I as well as C, N, and H atoms from the LD perovskitoids are colored in dark gray, purple, brown, light blue, and light gray, respectively. PbI_6 octahedra are highlighted.

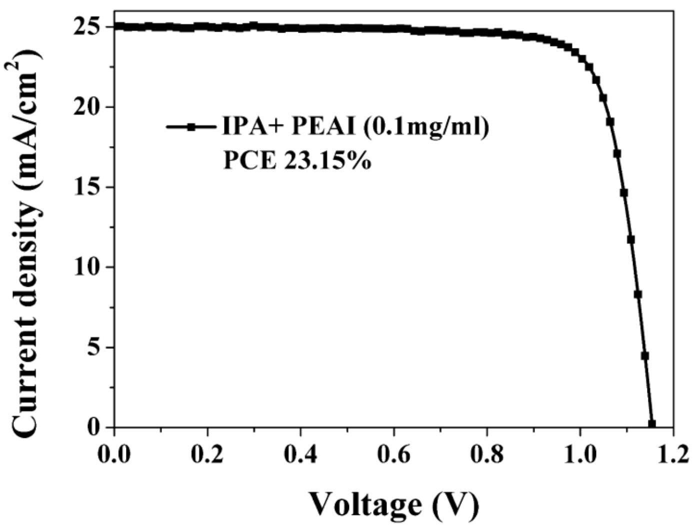

Fig. S12. *J-V* curve of PSC. The *J-V* curve of IPA+PEAI treated PSCs.

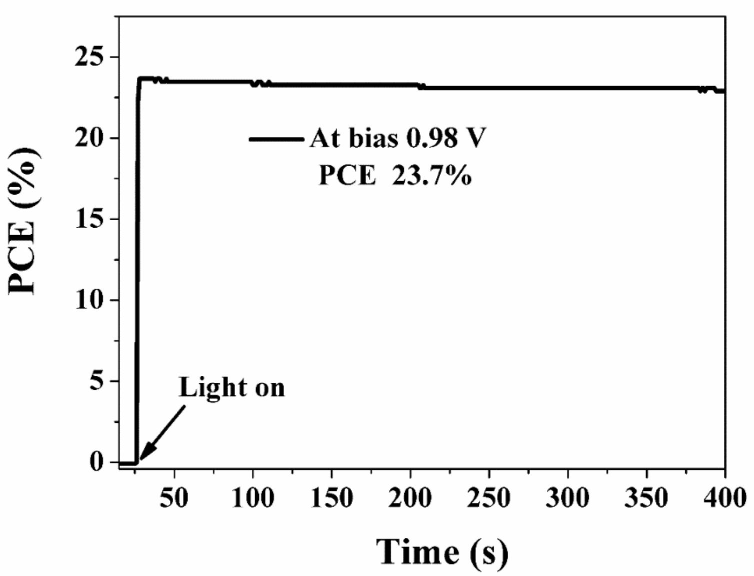

Fig. S13. Steady-state output of PSC. Steady-state photocurrent density and power output at maximum power point for 3D/0D device.

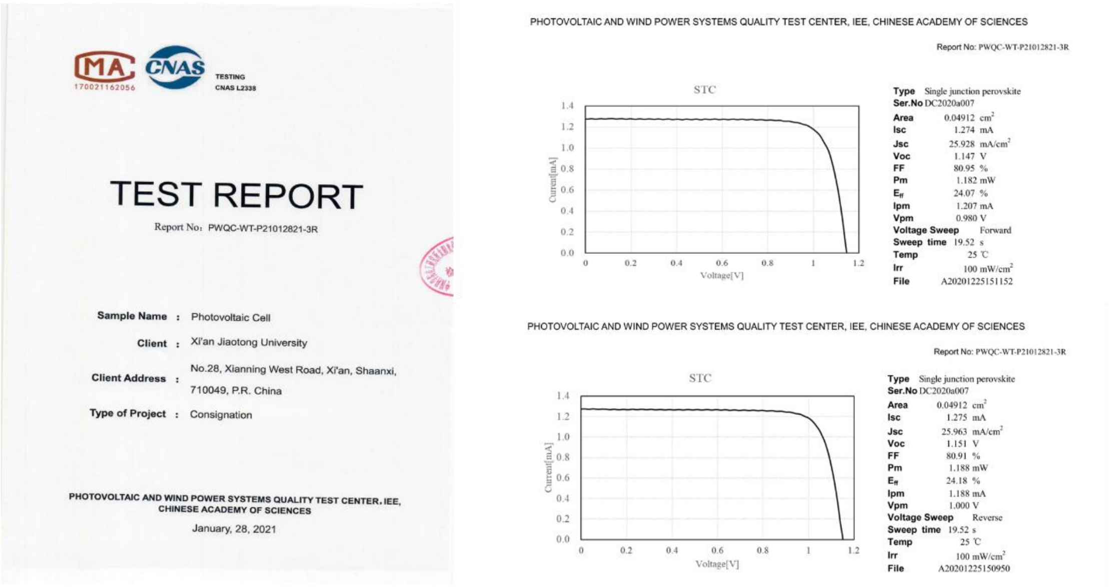

Fig. S14. Independent efficiency test of PSC. Independent efficiency test of perovskite solar cells by an accredited institute of the Photovoltaic and Wind Power Systems Quality Test Center (Chinese Academy of Science, China). The sample were tested under 40% humidity and 25 ℃ without encapsulation.

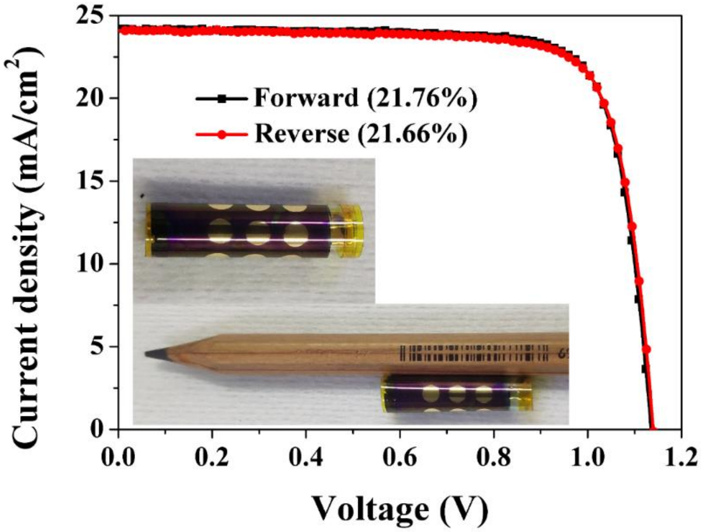

Fig. S15. *J*-*V* curve of PSC. The *J*-*V* curve of flexible device prepared with 0.1 mg/ml IPA + *m*-$PBAI_2$. Photo credit Jinbo chen, Xi'an Jiaotong University.

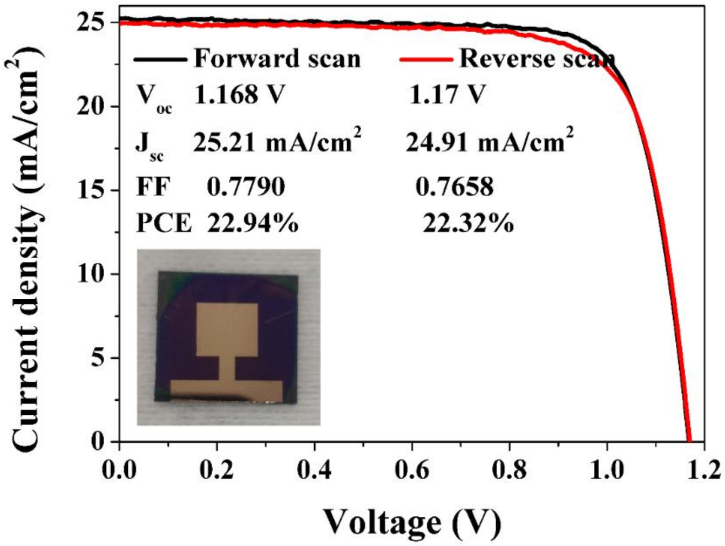

Fig. S16. *J*-*V* curve of PSC. The *J*-*V* curve of 1 cm^2 device prepared with 0.1 mg/ml IPA+*m*-$PBAI_2$. Photo credit Jinbo chen, Xi'an Jiaotong University.

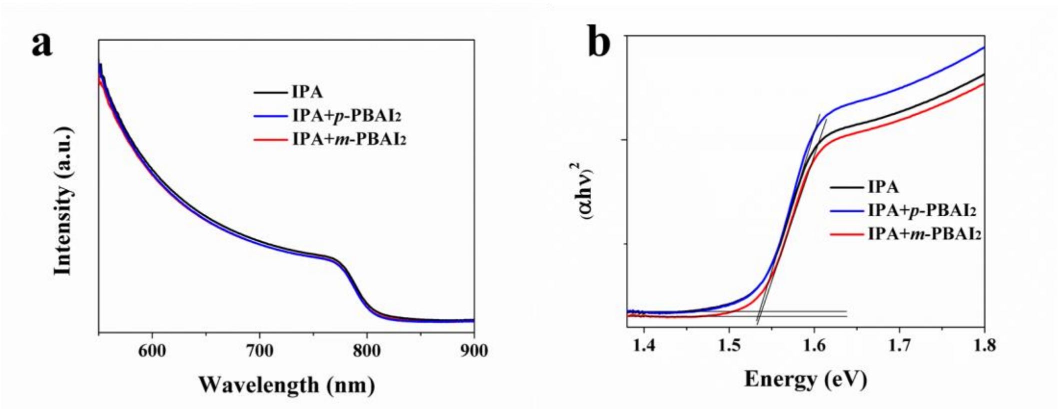

Fig. S17. Bandgap for different perovskite films. (a) The UV-vis absorption spectra of different perovskite films. (b) Tauc plots for bandgap estimation of different perovskite films.

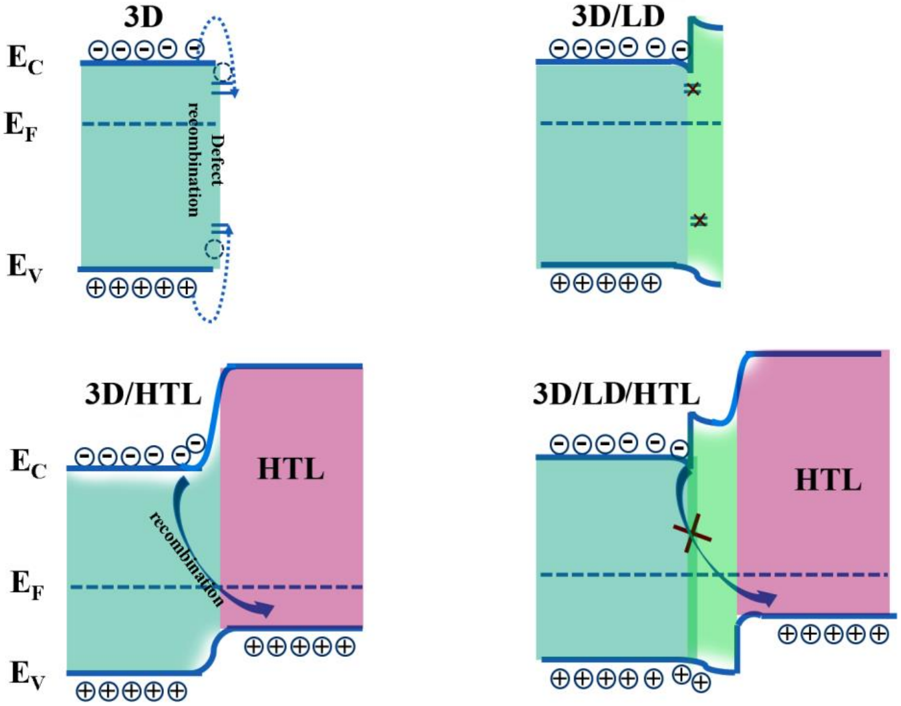

Fig. S18. Energy-band diagrams. Energy-band diagrams of perovskite film and perovskite/hole transport layer (HTL) junctions. E_C, E_F, and E_V stand for the conduction-band edge, Fermi level, and valence-band edge, respectively.

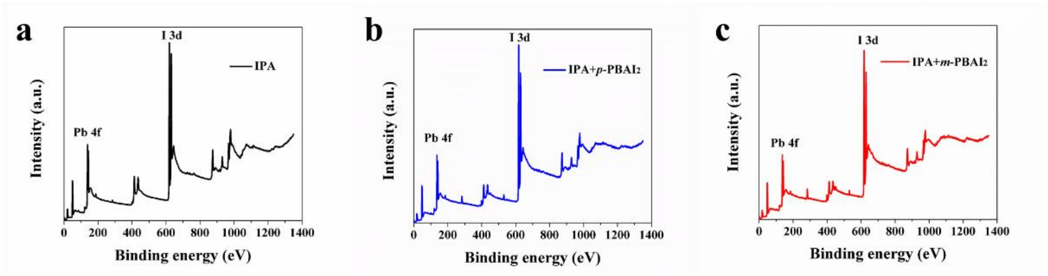

Fig. S19. XPS spectra for perovskite films. XPS spectra of perovskite prepared with pure IPA, IPA+*p*-$PBAI_2$, IPA+*m*-$PBAI_2$.

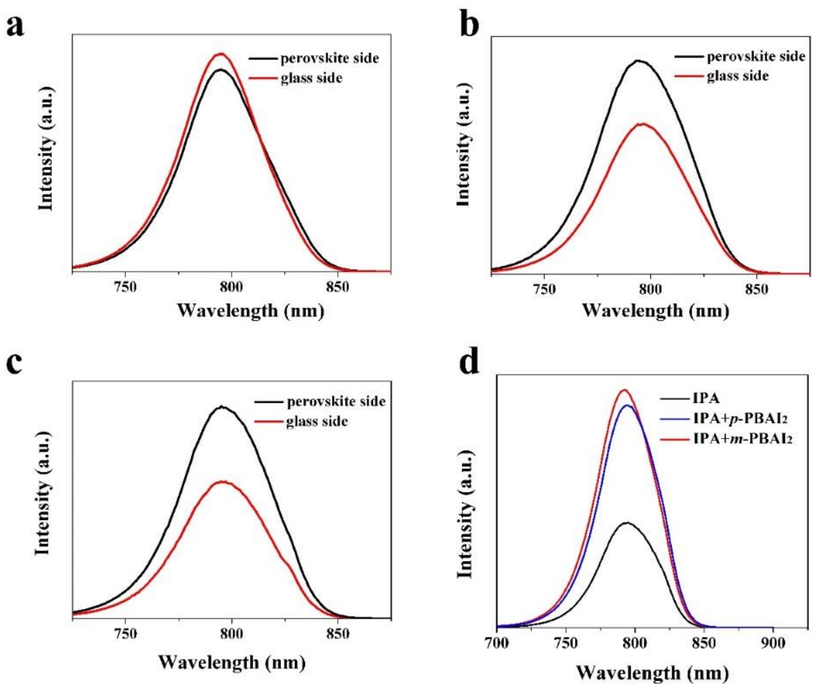

Fig. S20. Steady-state PL spectra of different perovskite films with different excitation directions. (a) pure 3D perovskite film, (b) IPA + *p*-$PBAI_2$ film, (c) IPA + *m*-$PBAI_2$ film, (d) all investigated films excited from the 3D perovskite side.

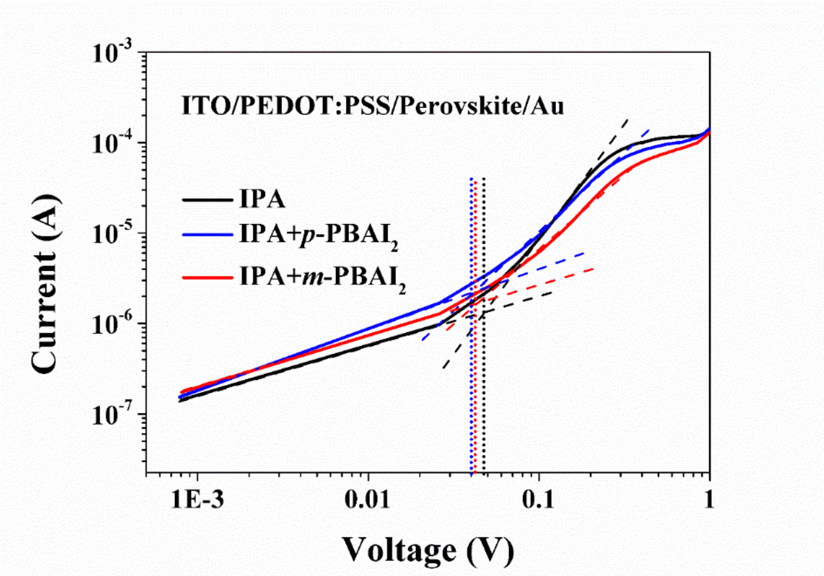

Fig. S21. SCLC measurements. Logarithm of *J* – *V* curves in the dark for IPA, IPA + *p*-$PBAI_2$, and IPA + *m*-$PBAI_2$ hole-only devices. The applied voltage at the first kink point is defined as the trapfilled limit voltage (V_{TFL}), which is determined by the trap state density (N_t) as can be seen in Equation: $V_{TFL} = \frac{eN_t d^2}{2\varepsilon_0 \varepsilon}$ where d is the film thickness, ε is the relative dielectric constantfor perovskite film (for $FAMAPbI_3$, $\varepsilon \approx 62.23$), and ε_0 (8.85×10^{-12}) is the constant of permittivity in free space.

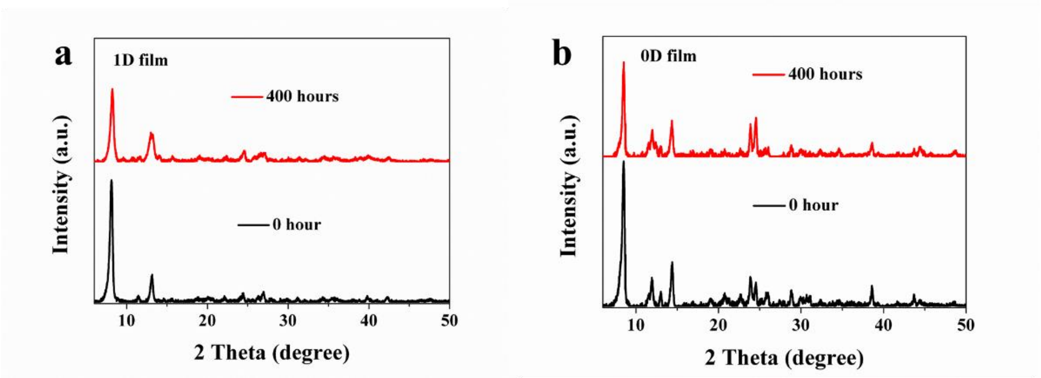

Fig. S22. XRD patterns of perovskite films. The XRD patterns of the 1D (a) and 0D (b) film before and after ageing at 85 ℃ /85% relative-humidity for 400 hours.

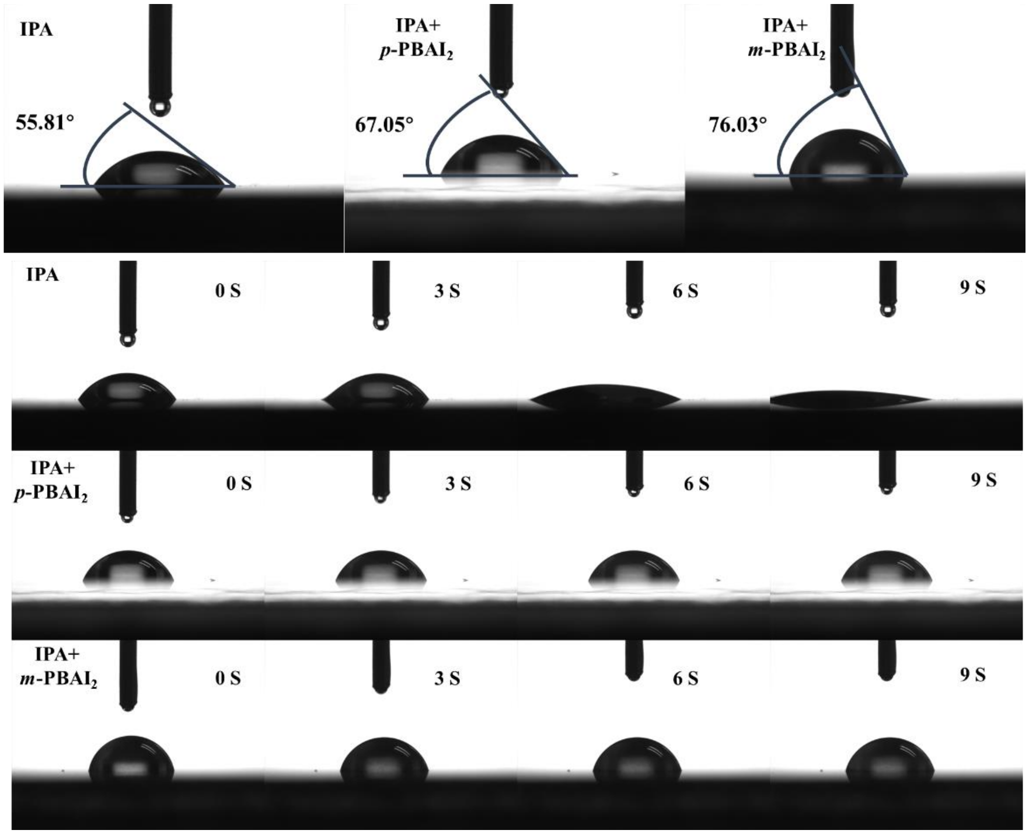

Fig. S23. Water contact test of perovskite films. Water contact angle of perovskite film based on IPA, IPA + *p*-$PBAI_2$, and IPA + *m*-$PBAI_2$, respectively. Time-dependent contact angle measurements show that both 3D/1D and 3D/0D films exhibit better moisture resistance than pure 3D film.

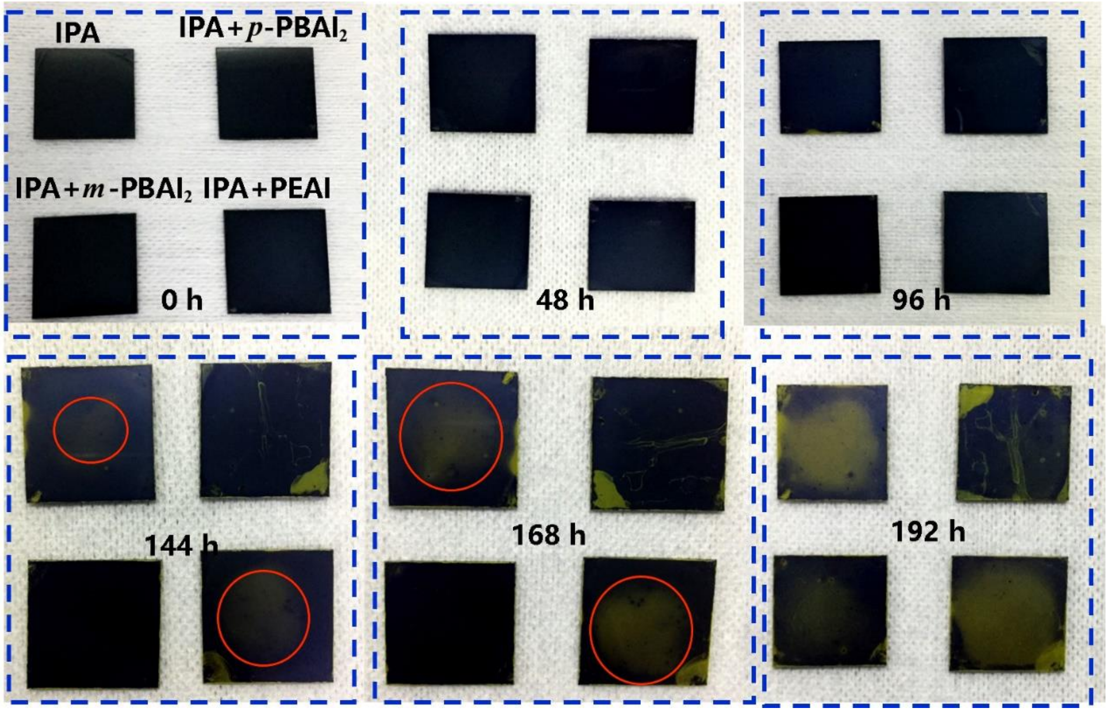

Fig. S24. Stability test of perovskite films. The graphic of perovskite film exposure at 85 ℃ under 85% humidity. Photo credit Jinbo chen, Xi'an Jiaotong University.

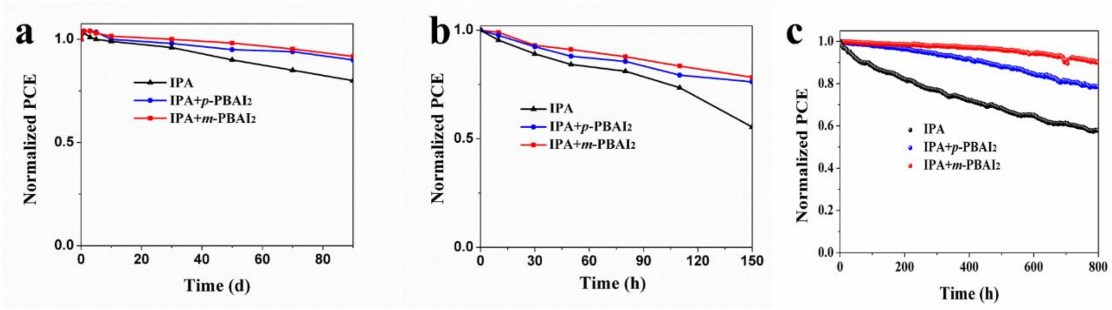

Fig. S25. Stability test of PSCs. (a) The stability test of different PSCs under 20% humidity and room temperature. (b) The stability test of different PSCs exposure at 85 ℃ under 85% humidity. (c) Under continuous light irradiation and MPP tracking with a white LED lamp at 100 mW cm^{-2} in air (30-40% humidity).

Table S1. The parameters of forward and reverse *J*-*V* scan of the best devices prepared with different method, hysteresis index (HI) are calculated with formula: HI $= \frac{PCE_{res} - PCE_{for}}{PCE_{res}}$.

Samples	Scanning direction	V_{oc} (V)	J_{sc} (mA/cm^2)	FF	PCE (%)	Hysteresis index
IPA	Forward	1.121	25.13	0.7717	21.74	0.0185
	Reverse	1.133	25.11	0.7786	22.15	
IPA + *p*-$PBAI_2$	Forward	1.148	25.67	0.8069	23.78	0.0025
	Reverse	1.155	25.52	0.8088	23.84	
IPA + *m*-$PBAI_2$	Forward	1.156	25.56	0.8207	24.25	0.0098
	Reverse	1.163	25.51	0.8254	24.49	

Table S2. Photovoltaic performance parameters for devices based on different *p*-$PBAI_2$ concentration in IPA.

	V_{oc} (V)	J_{sc} (mA/cm^2)	FF	PCE (%)
IPA	1.126 ±0.004 (1.129)	25.29 ±0.199 (25.42)	0.7742 ±0.007 (0.7836)	22.05 ±0.332 (22.49)
0.05 mg/ml	1.147 ±0.007 (1.16)	25.41 ±0.204 (25.45)	0.7615 ±0.011 (0.7725)	22.20 ±0.427 (22.81)
0.1 mg/ml	1.158 ±0.006 (1.155)	25.52 ±0.246 (25.52)	0.7859 ±0.011 (0.8088)	23.24 ±0.363 (23.84)
0.15 mg/ml	1.146 ±0.009 (1.163)	25.35 ±0.240 (25.39)	0.7653 ±0.009 (0.7813)	22.24 ±0.376 (23.07)

Table S3. Photovoltaic performance parameters for devices based on different *m*-$PBAI_2$ concentration in IPA.

	V_{oc} (V)	J_{sc} (mA/cm^2)	FF	PCE (%)
IPA	1.126 ±0.007 (1.129)	25.29 ±0.195 (25.42)	0.7742 ±0.011 (0.7836)	22.05 ±0.405 (22.49)
0.05 mg/ml	1.147 ±0.006 (1.157)	25.44 ±0.193 (25.63)	0.7679 ±0.012 (0.7894)	22.41 ±0.428 (23.41)
0.1 mg/ml	1.162 ±0.005 (1.163)	25.53 ±0.188 (25.51)	0.7899 ±0.017 (0.8255)	23.45 ±0.488 (24.49)
0.15 mg/ml	1.149 ±0.007 (1.158)	25.43 ±0.162 (25.52)	0.7656 ±0.013 (0.7843)	22.39 ±0.457 (23.18)

Table S4. Photovoltaic performance parameters for devices based on 0.1 mg/ml IPA + *m*-$PBAI_2$.

	V_{oc} (V)	J_{sc} (mA/cm^2)	FF	PCE (%)
1	1.166	25.80	0.7971	23.98
2	1.168	25.56	0.7892	23.56
3	1.163	25.54	0.7818	23.22
4	1.165	25.28	0.7917	23.32
5	1.169	25.45	0.7859	23.38
6	1.168	25.35	0.7904	23.40
7	1.163	25.90	0.7783	23.44
8	1.158	25.84	0.7865	23.53
9	1.16	25.61	0.7806	23.19
10	1.164	25.78	0.7736	23.22
11	1.164	24.98	0.7698	22.38
12	1.168	25.60	0.7715	23.06
13	1.158	25.38	0.8067	23.71
14	1.159	25.41	0.7821	23.03
15	1.161	25.65	0.7819	23.29
16	1.159	25.52	0.7868	23.27
17	1.159	25.48	0.7772	22.96
18	1.164	25.43	0.7831	23.18
19	1.163	25.76	0.7915	23.71
20	1.164	25.54	0.7842	23.32
21	1.157	25.58	0.7858	23.25
22	1.169	25.73	0.7820	23.52
23	1.154	25.26	0.8206	23.92
24	1.168	25.61	0.7851	23.49
25	1.166	25.73	0.7905	23.71
26	1.168	25.71	0.7895	23.71
27	1.163	25.76	0.7842	23.50
28	1.163	25.39	0.7813	23.07
29	1.168	25.60	0.7712	23.06
30	1.157	25.40	0.8225	24.17
31	1.156	25.38	0.8051	23.62
32	1.165	25.51	0.8119	24.13
33	1.163	25.51	0.8255	24.49
34	1.168	25.50	0.7567	22.54
35	1.169	25.28	0.7588	22.42
36	1.148	25.70	0.8172	24.11
37	1.15	25.39	0.8137	23.76
38	1.169	25.39	0.7741	22.98
39	1.161	25.48	0.8225	24.33
40	1.168	25.26	0.7715	22.76
41	1.163	25.39	0.8229	24.30
42	1.168	25.82	0.7968	24.03
43	1.164	25.45	0.7867	23.31
Average	1.163	25.53	0.7899	23.45

Table S5. Photovoltaic performance parameters for devices based on 0.1 mg/ml IPA + *p*-PBAI$_2$.

	V_{oc} (V)	J_{sc} (mA/cm^2)	FF	PCE (%)
1	1.16	25.34	0.7636	22.45
2	1.162	25.01	0.8076	23.47
3	1.163	25.02	0.7748	22.54
4	1.163	25.61	0.7789	23.20
5	1.161	25.43	0.7963	23.51
6	1.16	24.98	0.7732	22.41
7	1.157	25.5	0.7999	23.60
8	1.166	25.26	0.7762	22.86
9	1.162	25.48	0.7736	22.91
10	1.159	25.15	0.7669	22.35
11	1.161	25.15	0.7739	22.60
12	1.157	25.24	0.7655	22.36
13	1.158	25.69	0.7692	22.88
14	1.157	25.69	0.7639	22.70
15	1.164	25.78	0.7888	23.67
16	1.155	25.33	0.8087	23.66
17	1.155	25.52	0.8088	23.84
18	1.174	25.17	0.7598	22.45
19	1.166	24.94	0.7638	22.21
20	1.161	25.36	0.7760	22.85
21	1.149	25.6	0.7996	23.52
22	1.152	25.65	0.7834	23.15
23	1.143	25.69	0.7972	23.41
24	1.151	25.22	0.7937	23.04
25	1.153	25.84	0.7874	23.46
26	1.158	25.76	0.7895	23.55
27	1.164	25.30	0.7921	23.33
28	1.159	25.45	0.7912	23.34
29	1.161	25.60	0.7862	23.36
30	1.165	25.63	0.7912	23.63
31	1.159	25.63	0.7892	23.45
32	1.163	25.58	0.7808	23.23
33	1.166	25.82	0.7847	23.62
34	1.166	25.76	0.7850	23.58
35	1.166	25.84	0.7803	23.51
36	1.155	25.20	0.7764	22.60
37	1.165	25.61	0.7804	23.29
38	1.149	25.12	0.8232	23.76
39	1.159	25.67	0.7600	22.61
40	1.16	25.72	0.7828	23.36
41	1.16	25.47	0.7802	23.05
42	1.162	25.26	0.7878	23.12
43	1.159	25.20	0.7850	22.93
Average	1.158	25.52	0.7859	23.24

Table S6. Photovoltaic performance parameters for devices based on pure IPA.

	V_{oc} (V)	J_{sc} (mA/cm^2)	FF	PCE (%)
1	1.129	25.43	0.7836	22.50
2	1.129	25.35	0.7841	22.44
3	1.126	25.33	0.7855	22.40
4	1.123	25.78	0.7720	22.35
5	1.124	25.33	0.7739	22.03
6	1.133	25.27	0.7699	22.04
7	1.125	25.16	0.7750	21.94
8	1.128	25.18	0.7785	22.11
9	1.132	25.27	0.7742	22.15
10	1.131	25.53	0.7752	22.39
11	1.131	25.39	0.7825	22.47
12	1.127	25.27	0.7822	22.28
13	1.125	25.44	0.7861	22.50
14	1.124	25.19	0.7700	21.80
15	1.126	25.00	0.7705	21.69
16	1.122	25.47	0.7656	21.88
17	1.12	25.19	0.7662	21.62
18	1.119	25.30	0.7720	21.86
19	1.12	25.61	0.7644	21.93
20	1.129	25.39	0.7609	21.81
21	1.131	25.43	0.7776	22.36
22	1.122	25.33	0.7822	22.23
23	1.127	25.26	0.7764	22.10
24	1.133	25.12	0.7722	21.98
25	1.126	25.15	0.7736	21.90
26	1.131	25.43	0.7810	22.46
27	1.12	25.19	0.7686	21.68
28	1.132	25.00	0.7787	22.04
29	1.129	24.76	0.7671	21.44
30	1.119	25.00	0.7560	21.15
Average	1.126	25.28	0.7742	22.05

Table S7. Photovoltaic performance parameters for flexible devices based on 0.1 mg/ml IPA+*m*-$PBAI_2$.

Flexible	V_{oc} (V)	J_{sc} (mA/cm^2)	FF	PCE (%)
Forward scan	1.135	24.19	0.7926	21.76
Reverse scan	1.139	24.08	0.7897	21.66

Table S8. The TRPL fitting parameters of different perovskite film.

Sample	τ_1 (ns)	A_1 (%)	τ_2 (ns)	A_2 (%)	τ_{ave} (ns)
IPA	126.06	23.74	821.21	76.26	656.18
IPA+*p*-$PBAI_2$	156.08	18.22	1134.36	81.78	956.11
IPA+*m*-$PBAI_2$	208.66	13.56	1337.88	86.44	1184.76

REFERENCES AND NOTES

1. N. J. Jeon, J. H. Noh, Y. C. Kim, W. S. Yang, S. Ryu, S. I. Seok, Solvent engineering for high-performance inorganic–organic hybrid perovskite solar cells. *Nat. Mater.* **13**, 897–903 (2014).

2. M. M. Lee, J. Teuscher, T. Miyasaka, T. N. Murakami, H. J. Snaith, Efficient hybrid solar cells based on meso-superstructured organometal halide perovskites. *Science* **338**, 643–647 (2012).

3. S. D. Stranks, G. E. Eperon, G. Grancini, C. Menelaou, M. J. P. Alcocer, T. Leijtens, L. M. Herz, A. Petrozza, H. J. Snaith, Electron-hole diffusion lengths exceeding 1 micrometer in an organometal trihalide perovskite absorber. *Science* **342**, 341–344 (2013).

4. X. Li, D. Bi, C. Yi, J.-D. Décoppet, J. Luo, S. M. Zakeeruddin, A. Hagfeldt, M. Grätzel, A vacuum flash-assisted solution process for high-efficiency large-area perovskite solar cells. *Science* **353**, 58–62 (2016).

5. H. Tsai, R. Asadpour, J. C. Blancon, C. C. Stoumpos, O. Durand, J. W. Strzalka, B. Chen, R. Verduzco, P. M. Ajayan, S. Tretiak, J. Even, M. A. Alam, M. G. Kanatzidis, W. Nie, A. D. Mohite, Light-induced lattice expansion leads to high-efficiency perovskite solar cells. *Science* **360**, 67–70 (2018).

6. W.-J. Yin, T. Shi, Y. Yan, Unusual defect physics in $CH_3NH_3PbI_3$ perovskite solar cell absorber. *Appl. Phys. Lett.* **104**, 063903 (2014).

7. K. X. Steirer, P. Schulz, G. Teeter, V. Stevanovic, M. Yang, K. Zhu, J. J. Berry, Defect tolerance in methylammonium lead triiodide perovskite. *ACS Energy Lett.* **1**, 360–366 (2016).

8. A. Walsh, D. O. Scanlon, S. Y. Chen, X. G. Gong, S. H. Wei, Self-regulation mechanism for charged point defects in hybrid halide perovskites. *Angew. Chem. Int. Ed.* **54**, 1791–1794 (2015).

9. J. Kim, S. H. Lee, J. H. Lee, K. H. Hong, The role of intrinsic defects in methylammonium lead iodide perovskite. *J. Phys. Chem. Lett.* **5**, 1312–1317 (2014).

10. J. M. Ball, A. Petrozza, Defects in perovskite-halides and their effects in solar cells. *Nat. Energy* **1**, 16149 (2016).

11. X. Wu, M. T. Trinh, D. Niesner, H. Zhu, Z. Norman, J. S. Owen, O. Yaffe, B. J. Kudisch, X.-Y. Zhu, Trap states in lead iodide perovskites. *J. Am. Chem. Soc.* **137**, 2089–2096 (2015).

12. R. Long, J. Liu, O. V. Prezhdo, Unravelling the effects of grain boundary and chemical doping on electron–hole recombination in $CH_3NH_3PbI_3$ perovskite by time-domain atomistic simulation. *J. Am. Chem. Soc.* **138**, 3884–3890 (2016).

13. Y. Shao, Z. Xiao, C. Bi, Y. Yuan, J. Huang, Origin and elimination of photocurrent hysteresis by fullerene passivation in $CH_3NH_3PbI_3$ planar heterojunction solar cells. *Nat. Commun.* **5**, 5784 (2014).

14. J. Xu, A. Buin, A. H. Ip, W. Li, O. Voznyy, R. Comin, M. Yuan, S. Jeon, Z. Ning, J. J. McDowell, P. Kanjanaboos, J.-P. Sun, X. Lan, L. N. Quan, D. H. Kim, I. G. Hill, P. Maksymovych, E. H. Sargent, Perovskite–fullerene hybrid materials suppress hysteresis in planar diodes. *Nat. Commun.* **6**, 7081 (2015).

15. Y. Lin, B. Chen, F. Zhao, X. Zheng, Y. Deng, Y. Shao, Y. Fang, Y. Bai, C. Wang, J. Huang, Matching charge extraction contact for wide-bandgap perovskite solar cells. *Adv. Mater.* **29**, 1700607 (2017).

16. T. Niu, J. Lu, M. Tang, D. Barrit, D. Smilgies, Z. Yang, J. Li, Y. Fan, T. Luo, I. McCulloch, A. Amassian, S. Liu, K. Zhao, High performance ambient-air-stable $FAPbI_3$ perovskite solar cells with molecule-passivated Ruddlesden-Popper/3D heterostructured film. *Energy Environ. Sci.* **11**, 3358–3366 (2018).

17. S. G. Kim, J. Chen, J.-Y. Seo, D.-H. Kang, N.-G. Park, Rear-surface passivation by melaminium iodide additive for stable and hysteresis-less perovskite solar cells. *ACS Appl. Mater. Interfaces* **10**, 25372–25383 (2018).

18. D. W. deQuilettes, S. Koch, S. Burke, R. K. Paranji, A. J. Shropshire, M. E. Ziffer, D. S. Ginger, Photoluminescence lifetimes exceeding 8 μs and quantum yields exceeding 30% in hybrid perovskite thin films by ligand passivation. *ACS Energy Lett.* **1**, 438–444 (2016).

19. B. Wang, F. Wu, S. Bi, J. Zhou, J. Wang, X. Leng, D. Zhang, R. Meng, B. Xue, C. Zong, L. Zhu, Y. Zhang, H. Zhou, A polyaspartic acid sodium interfacial layer enhances surface trap passivation in perovskite solar cells. *J. Mater. Chem. A* **7**, 23895–23903 (2019).

20. E. A. Alharbi, A. Y. Alyamani, D. J. Kubicki, A. R. Uhl, B. J. Walder, A. Q. Alanazi, J. Luo, A. B. Caminal, A. Albadri, H. Albrithen, M. H. Alotaibi, J. E. Moser, S. M. Zakeeruddin, F. Giordano, L. Emsley, M. Grätzel, Atomic-level passivation mechanism of ammonium salts enabling highly efficient perovskite solar cells. *Nat. Commun.* **10**, 3008 (2019).

21. Z. Wang, Q. Lin, F. P. Chmiel, N. Sakai, L. M. Herz, H. J. Snaith, Efficient ambient-air-stable solar cells with 2D-3D heterostructured butylammonium-caesium-formamidinium lead halide perovskites. *Nat. Energy* **2**, 17135 (2017).

2. E. Jokar, C. Chien, A. Fathi, M. Rameez, Y. Chang, E. W.-G. Diau, Efficient ambient-air-stable solar cells with 2D-3D heterostructured butylammonium-caesium-formamidinium lead halide perovskites. *Energy Environ. Sci.* **11**, 2353–2362 (2018).

23. Q. Jiang, Y. Zhao, X. Zhang, X. Yang, Y. Chen, Z. Chu, Q. Ye, X. Li, Z. Yin, J. You, Surface passivation of perovskite film for efficient solar cells. *Nat. Photonics* **13**, 460–466 (2019).

24. J. Xi, J. Byeon, U. Kim, K. Bang, G. R. Han, J.-Y. Kim, J. Yoon, H. Dong, Z. Wu, G. Divitini, K. Xi, J. Park, T.-W. Lee, S. K. Kim, M. Choi, J. W. Lee, Abnormal spatial heterogeneity governing the charge-carrier mechanism in efficient Ruddlesden-Popper perovskite solar cells. *Energy Environ. Sci.* **14**, 4915–4925 (2021).

25. Y. Cho, A. Soufiani, J. Yun, J. Kim, D. Lee, J. Seidel, X. Deng, M. A. Green, S. Huang, A. W. Y. Ho-Baillie, Mixed 3D-2D passivation treatment for mixed-cation lead mixed-halide perovskite solar cells for higher efficiency and better stability. *Adv. Energy Mater.* **8**, 1703392 (2018).

26. C. C. Stoumpos, L. Mao, C. D. Malliakas, M. G. Kanatzidis, Structure–band gap relationships in hexagonal polytypes and low-dimensional structures of hybrid tin iodide perovskites. *Inorg. Chem.* **56**, 56–73 (2017).

27. A. Marronnier, G. Roma, S. Boyer-Richard, L. Pedesseau, J.-M. Jancu, Y. Bonnassieux, C. Katan, C. C. Stoumpos, M. G. Kanatzidis, J. Even, An harmonicity and disorder in the black phases of cesium lead iodide used for stable inorganic perovskite solar cells. *ACS Nano* **12**, 3477–3486 (2018).

28. Q. Sun, Y. Xu, H. Zhang, B. Xiao, X. Liu, J. Dong, Y. Cheng, B. Zhang, W. Jie, M. G. Kanatzidis, Optical and electronic anisotropies in perovskitoid crystals of Cs3Bi2I9 studies of nuclear radiation detection. *J. Mater. Chem. A* **6**, 23388–23395 (2018).

29. W. Lin, J. He, K. M. McCall, C. C. Stoumpos, Z. Liu, I. Hadar, S. Das, H.-H. Wang, B.-X. Wang, D. Y. Chung, B. W. Wessels, M. G. Kanatzidis, Inorganic halide perovskitoid $TlPbI_3$ for ionizing radiation detection. *Adv. Funct. Mater.* **31**, 2006635 (2021).

30. X. Li, Y. He, M. Kepenekian, P. Guo, W. Ke, J. Even, C. Katan, C. C. Stoumpos, R. D. Schaller, M. G. Kanatzidis, Three-dimensional lead iodide perovskitoid hybrids with high x-ray photoresponse. *J. Am. Chem. Soc.* **142**, 6625–6637 (2020).

31. T. Kong, H. Xie, Y. Zhang, J. Song, Y. Li, E. L. Lim, A. Hagfeldt, D. Bi, Perovskitoid-templated formation of a 1D@3D perovskite structure toward highly efficient and stable perovskite solar cells. *Adv. Energy Mater.* **11**, 2101018 (2021).

32. P. Kour, M. C. Reddy, S. Pal, S. Sidhik, T. Das, P. Pandey, S. P. Mukherjee, S. Chakraborty, A. D. Mohite, S. Ogale, An organic-inorganic perovskitoid with zwitterion cysteamine linker and its crystal-crystal transformation to Ruddlesden-Popper phase. *Angew. Chem. Int. Ed.* **60**, 18750–18760 (2021).

33. M. Daub and H. Hillebrecht, From 1D to 3D: Perovskites within the system $HSC(NH_2)_2I/CH_3NH_3I/PbI_2$ with maintenance of the cubic closest packing. *Inorg. Chem.* **60**, 3082–3093 (2021).

34. M. Wilke and N. Casati, Insight into the mechanochemical synthesis and structural evolution of hybrid organic-inorganic guanidinium lead(II) iodides. *Chem. Eur. J.* **24**, 17701–17711 (2018).

35. B. Park, N. Kedem, M. Kulbak, D. Y. Lee, W. S. Yang, N. J. Jeon, J. Seo, G. Kim, K. J. Kim, T. J. Shin, G. Hodes, D. Cahen, S. I. Seok, Understanding how excess lead iodide precursor improves halide perovskite solar cell performance. *Nat. Commun.* **9**, 3301 (2018).

36. Y. Yang, S. Feng, M. Li, F. Li, C. Zhang, Y. Han, L. Li, J. Yu, L. Cao, Z. Wang, B. Sun, X. Gao, Enormously improved $CH_3NH_3PbI_3$ film surface for environmentally stable planar perovskite solar cells with PCE exceeding 19.9%. *Nano Energy* **48**, 10–19 (2018).

37. Y. Liu, S. Akin, L. Pan, R. Uchida, N. Arora, J. V. Milić, A. Hinderhofer, F. Schreiber, A. R. Uhl, S. M. Zakeeruddin, A. Hagfeldt, M. I. Dar, M. Grätzel, Ultrahydrophobic 3D/2D fluoroarene bilayer-based water-resistant perovskite solar cells with efficiencies exceeding 22%. *Sci. Adv.* **5**, eaaw2543 (2019).

38. C. Eames, J. M. Frost, P. R.F. Barnes, B. C. O'Regan, A. Walsh, M. S. Islam, Ionic transport in hybrid lead iodide perovskite solar cells. *Nat. Commun.* **6**, 7497 (2015).

39. J. H. Heo, H. J. Han, D. Kim, T. K. Ahn, S. H. Im, Hysteresis-less inverted $CH_3NH_3PbI_3$ planar perovskite hybrid solar cells with 18.1% power conversion efficiency. *Energy Environ. Sci.* **8**, 1602–1608 (2015).

40. D. Yang, X. Zhou, R. Yang, Z. Yang, W. Yu, X. Wang, C. Li, S. Liu, R. P. H. Chang, Surface optimization to eliminate hysteresis for record efficiency planar perovskite solar cells. *Energy Environ. Sci.* **9**, 3071–3078 (2016).

41. J. Zhuang, P. Mao, Y. Luan, X. Yi, Z. Tu, Y. Zhang, Y. Yi, Y. Wei, N. Chen, T. Lin, F. Wang, C. Li, J. Wang, Interfacial passivation for perovskite solar cells: The effects of the functional group in phenethylammonium iodide. *ACS Energy Lett.* **4**, 2913–2921 (2019).

42. S. Yang, S. Chen, E. Mosconi, Y. Fang, X. Xiao, C. Wang, Y. Zhou, Z. Yu, J. Zhao, Y. Gao, F. D. Angelis, J. Huang, Stabilizing halide perovskite surfaces for solar cell operation with wide-bandgap lead oxysalts. *Science* **365**, 473–478 (2019).

43. X. Yi, Y. Mao, L. Zhang, J. Zhuang, Y. Zhang, N. Chen, T. Lin, Y. Wei, F. Wang, J. Wang, C. Li, Enhanced optoelectronic quality of perovskite thin films with hypophosphorous acid for planar heterojunction solar cells. *Small Methods* **6**, 2000441 (2020).

44. W. Zhang, S. Pathak, N. Sakai, T. Stergiopoulos, P. K. Nayak, N. K. Noel, A. A. Haghighirad, V. M. Burlakov, D. W. deQuilettes, A. Sadhanala, W. Li, L. Wang, D. S Ginger, R. H. Friend, H. J. Snaith, Enhanced optoelectronic quality of perovskite thin films with hypophosphorous acid for planar heterojunction solar cells. *Nat. Commun.* **6**, 10030 (2015).

45. R. Lindblad, D. Bi, B.-W. Park, J. Oscarsson, M. Gorgoi, H. Siegbahn, M. Odelius, E. M. J. Johansson, H. Rensmo, Electronic structure of $TiO_2/CH_3NH_3PbI_3$ perovskite solar cell interfaces. *J. Phys. Chem. Lett.* **5**, 648–653 (2014).

46. W. S. Yang, B.-W. Park, E. H. Jung, N. J. Jeon, Y. C. Kim, D. U. Lee, S. S. Shin, J. Seo, E. K. Kim, J. H. Noh, S. I. Seok, Iodide management in formamidinium-lead-halide–based perovskite layers for efficient solar cells. *Science* **356**, 1376–1379 (2017).

47. S. Chen, X. Xiao, H. Gu, J. Huang, Iodine reduction for reproducible and high-performance perovskite solar cells and modules. *Sci. Adv.* **7**, eabe8130 (2021).

48. W. Chen, Y. Wu, J. Fan, A. B. Djurišic, F. Liu, H. W. Tam, A. Ng, C. Surya, W. K. Chan, D. Wang, Z.-B. He, Understanding the doping effect on NiO: Toward high-performance inverted perovskite solar cells. *Adv. Energy Mater.* **8**, 1703519 (2018).

49. S. R. Cowan, A. Roy, A. J. Heeger, Recombination in polymer-fullerene bulk heterojunction solar cells. *Phys. Rev. B* **82**, 245207 (2010).

50. J. W. Jung, C.-C. Chueh, A. K.-Y. Jen, High-performance semitransparent perovskite solar cells with 10% power conversion efficiency and 25% average visible transmittance based on transparent CuSCN as the hole-transporting material. *Adv. Energy Mater.* **5**, 1500486 (2015).

51. J. Duan, Y. Zhao, B. He, Q. Tang, High-purity inorganic perovskite films for solar cells with 9.72% efficiency. *Angew. Chem. Int. Ed.* **57**, 3787–3791 (2018).

52. J. Xi, I. Spanopoulos, K. Bang, J. Xu, H. Dong, Y. Yang, C. D. Malliakas, J. M. Hoffman, M. G. Kanatzidis, Z. Wu, Alternative organic spacers for more efficient perovskite solar cells containing Ruddlesden-Popper phases, *J. Am. Chem. Soc.* **46**, 19705–19714 (2020).

53. G. Lv, L. Li, D. Lu, Z. Xu, Y. Dong, Q. Li, Z. Chang, W.-J. Yin, Y. Liu, Multiple-noncovalent-interaction-stabilized layered Dion-Jacobson perovskite for efficient solar cells. *Nano Lett.* **21**, 5788–5797 (2021).
